# Genome-wide identification of bacterial genes contributing to nucleus-forming jumbo phage infection

**DOI:** 10.1093/nar/gkae1194

**Published:** 2024-12-19

**Authors:** Kate R Harding, Lucia M Malone, Natalie A P Kyte, Simon A Jackson, Leah M Smith, Peter C Fineran

**Affiliations:** Department of Microbiology and Immunology, University of Otago, PO Box 56, Dunedin 9054, New Zealand; Genetics Otago, University of Otago, PO Box 56, Dunedin 9054, New Zealand; Maurice Wilkins Centre for Molecular Biodiscovery, University of Otago, PO Box 56, Dunedin 9054, New Zealand; Department of Microbiology and Immunology, University of Otago, PO Box 56, Dunedin 9054, New Zealand; Genetics Otago, University of Otago, PO Box 56, Dunedin 9054, New Zealand; Department of Microbiology and Immunology, University of Otago, PO Box 56, Dunedin 9054, New Zealand; Genetics Otago, University of Otago, PO Box 56, Dunedin 9054, New Zealand; Department of Microbiology and Immunology, University of Otago, PO Box 56, Dunedin 9054, New Zealand; Genetics Otago, University of Otago, PO Box 56, Dunedin 9054, New Zealand; Maurice Wilkins Centre for Molecular Biodiscovery, University of Otago, PO Box 56, Dunedin 9054, New Zealand; Bioprotection Aotearoa, University of Otago, PO Box 56, Dunedin 9054, New Zealand; Department of Microbiology and Immunology, University of Otago, PO Box 56, Dunedin 9054, New Zealand; Genetics Otago, University of Otago, PO Box 56, Dunedin 9054, New Zealand; Maurice Wilkins Centre for Molecular Biodiscovery, University of Otago, PO Box 56, Dunedin 9054, New Zealand; Department of Microbiology and Immunology, University of Otago, PO Box 56, Dunedin 9054, New Zealand; Genetics Otago, University of Otago, PO Box 56, Dunedin 9054, New Zealand; Maurice Wilkins Centre for Molecular Biodiscovery, University of Otago, PO Box 56, Dunedin 9054, New Zealand; Bioprotection Aotearoa, University of Otago, PO Box 56, Dunedin 9054, New Zealand

## Abstract

The *Chimalliviridae* family of bacteriophages (phages) form a proteinaceous nucleus-like structure during infection of their bacterial hosts. This phage ‘nucleus’ compartmentalises phage DNA replication and transcription, and shields the phage genome from DNA-targeting defence systems such as CRISPR-Cas and restriction-modification. Their insensitivity to DNA-targeting defences makes nucleus-forming jumbo phages attractive for phage therapy. However, little is known about the bacterial gene requirements during the infectious cycle of nucleus-forming phages or how phage resistance may emerge. To address this, we used the *Serratia* nucleus-forming jumbo phage PCH45 and exploited a combination of high-throughput transposon mutagenesis and deep sequencing (Tn-seq), and CRISPR interference (CRISPRi). We identified over 90 host genes involved in nucleus-forming phage infection, the majority of which were either involved in the biosynthesis of the primary receptor, flagella, or influenced swimming motility. In addition, the bacterial outer membrane lipopolysaccharide contributed to PCH45 adsorption. Other unrelated *Serratia*-flagellotropic phages used similar host genes as the nucleus-forming phage, indicating that phage resistance can lead to cross-resistance against diverse phages. Our findings demonstrate that resistance to nucleus-forming jumbo phages can readily emerge via bacterial surface receptor mutation and this should be a major factor when designing strategies for their use in phage therapy.

## Graphical Abstract

**Graphical Abstract:**
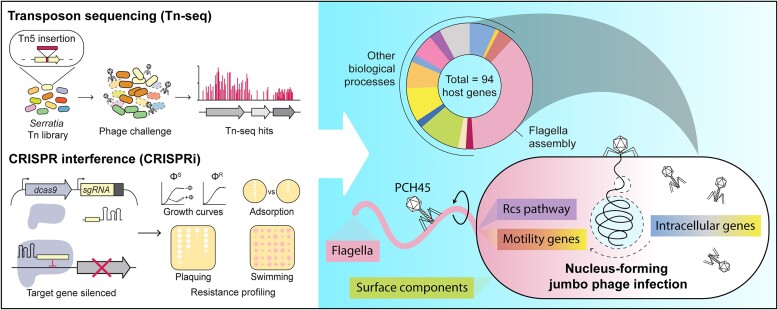

## Introduction

Bacterial viruses (phages) are abundant, present in diverse environments and exploit various strategies to infect their bacterial hosts (1–3). A diverse group of phages are designated as ‘jumbo’ due to their large double stranded (ds) DNA genomes of >200 kb (4,5). Due to their large genome, jumbo phages encode additional core and accessory genes compared with phages with smaller genomes (6) suggesting jumbo phages may use less host machinery for their replication and infection (7).

The *Chimalliviridae* viruses are jumbo phages that form a proteinaceous nucleus-like structure upon infection (8,9). Prior to the formation of the phage nucleus an ‘early phage infection’ (EPI) vesicle is rapidly formed to protect the injected phage genome and associated proteins before translocation to the phage nucleus (10). Within the phage nucleus, phage DNA replication and transcription occur, then transcripts are translocated to the cytoplasm for translation and thereafter phage assembly (8). The phage nucleus is primarily composed of multiple copies of the phage-encoded chimallin protein (ChmA), which self-assembles during the initial infection (9). Narrow pores are present within this chimallin shell as well as a phage-encoded nucleus-associated protein (ChmC), both of which may be involved in the transport of phage RNA to the host cytoplasm (9,11,12). However, the mechanism of RNA transport is not known. In φKZ, the import of phage or host proteins through the protein lattice of the phage nucleus is largely driven by Gp69 (known as PicA or Imp1) (13,14). The phage nucleus acts as a protective shield, preventing DNA-targeting bacterial defence systems, such as CRISPR-Cas and restriction modification, from accessing phage DNA (15,16).

Due to their lytic replication strategy and their ability to broadly evade DNA targeting defence systems, nucleus-forming phages may have an advantage over other phages when being used as an antimicrobial in phage therapies (8,15–17). Indeed, nucleus-forming phages infecting bacterial pathogens of agricultural, foodborne and clinical relevance are readily isolated (18–22). Importantly, jumbo phages have been employed for the successful treatment of a chronic extensively drug resistant *Pseudomonas* eye infection and acute hepatopancreatic necrosis disease caused by *Vibrio harveyi* in shrimps, highlighting their potential as anti-bacterial therapeutics (17,21,22).

Multiple studies have focused on understanding the molecular mechanisms of *Chimalliviridae* phage development and nucleus formation. Furthermore, the interactions between bacterial and nucleus-forming phage proteins, and how phage genes and proteins are involved in cell takeover is beginning to be addressed (11,23–25). For example, a φKZ protein interacts with the host RNA degradosome and prevents the degradation of viral RNA, whereas other phage proteins interact with ribosomes (23,26–28). Interestingly, *Chimalliviridae* proteins of unknown function also interact with bacterial proteins, but their involvement in host takeover is unknown (23). Whilst these studies provide a map of the phage-host interactome, they rely on direct interactions and overlook indirect pathways that may impact phage infection. Furthermore, understanding which mutations result in resistance to *Chimalliviridae* phages is essential for developing their use for phage therapy. The major advances in understanding *Chimalliviridae* infection are derived from *Pseudomonas* phages related to φKZ. To broaden our understanding of nucleus-forming phages it is critical to study the infection processes of different phage-host pairs.

Here we identified genetic loci contributing to nucleus-forming phage infection, using *Serratia* sp. ATCC 39006 (*Serratia*; or recently proposed naming as *Prodigiosinella confusarubida* (29)) and phage PCH45. We employed transposon mutagenesis and deep sequencing (Tn-seq), validated by CRISPRi knockdowns, to uncover non-essential bacterial loci involved in phage infection. By identifying the bacterial genes required for phage infection, we uncovered how a bacterium can evolve resistance to a nucleus-forming phage. Approximately half of the loci identified contributed to the biosynthesis and regulation of flagella receptor, with others involved in lipopolysaccharide (LPS) biosynthesis, regulation of transcription, signal transduction, amino acid synthesis and other biological processes. Overall, our findings show that nucleus-forming phages require diverse bacterial genes to support their successful infection. Despite their complex developmental strategy, nucleus-forming phages remain vulnerable to bacterial receptor mutation, and this should be a major consideration whilst developing these phages for therapy.

## Materials and methods

### Bacterial growth conditions

*Serratia* sp. ATCC 39006 (*Serratia*) (30) and *Escherichia coli* ST18 (*E. coli*) (31) (Supplementary Table S1) were grown in Lysogeny broth (LB; 10 g tryptone, 5 g yeast extract, 5 g NaCl, 1 L distilled water) at 30°C and 37°C with shaking (120 rpm). When grown on solid media, *Serratia* and *E. coli* were streaked onto Lysogeny broth agar (LBA; LB with 15 g agar in 1 L distilled water) (1.5% w/v) and incubated for 1 and 2 days respectively at an appropriate temperature, or until colony formation. When needed, antibiotic and supplements were added to the LB and LBA: chloramphenicol (Cm; 25 μg/ml), kanamycin (Km; 50 μg/ml), tetracycline (Tc), 5-aminolevulinic acid (ALA; 50 μg/ml) and arabinose (0.1% w/v).

### Phage stock preparation

Phages PCH45 (16), CHE70 (unpublished), JS26 (32), LC53 (33) and OT8 (34) stocks were prepared using the double agar overlay method (Supplementary Table S1). To determine the appropriate phage concentration, 100 μl of bacterial culture were mixed with 100 μl of 10-fold serial dilutions of phage lysate and 4 ml molten LBA overlay (0.35% w/v), poured onto an LBA plate and incubated at 30°C overnight. The dilution that produced webbed lysis (almost confluent lysis) was selected to prepare new phage stocks. Overlays of the desired phage dilution were scraped and pooled into a chloroform resistant centrifuge tube. Next, a few drops of chloroform (NaHCO_3_-saturated) were added and the samples were vortexed vigorously to lyse the cells. Cellular debris was removed by centrifugation (2000 *g* for 20 min) and the supernatant carrying the phage was transferred into a new tube and stored at 4°C. To calculate the phage titre, 10-fold serial dilutions were made in phage buffer (10 mM Tris–HCl pH 7.4, 10 mM MgSO_4_, 0.01% w/v gelatine) and spotted (10-20 μl) onto LBA overlay previously seeded with 100 μl of *Serratia* culture. After an overnight incubation at 30°C, plaques were counted, and phage titre was expressed as plaque forming units (PFU)/ml.

### Generation of saturated transposon mutant pool and phage challenge

The transposon saturated mutant pools were generated as described elsewhere (35). Briefly, an *E. coli* donor strain harbouring a Tn5 transposon delivery plasmid pKRCPN2, was grown overnight in LB supplemented with ALA and Tc. The *Serratia* LacA recipient strain (30), harbouring plasmid pPF781 was grown overnight in LB with Cm. All plasmids are listed in Supplementary Table S2. Cells were harvested through centrifugation (8000 *g* for 2 min), washed with LB and diluted to an OD_600_= 1. Equal volumes of each cultures were mixed, and 30 100 μl aliquots were spotted onto nitrocellulose filter paper (0.22 μl pore size) on LBA with ALA. Conjugation spots were incubated at 30°C overnight, and filters were collected in 15 ml of LB to resuspend cells. The cells were used to inoculate three flasks containing 500 ml of LB with Cm and Km at an OD_600_= 0.02 and incubated at 30°C with shaking (160 rpm) for 20 h, to select for *Serratia* transconjugants. Finally, 15 ml of each flask were pooled together, pelleted by centrifugation, and resuspended in LB with a final OD_600_= 3 generating a *Serratia* transposon mutant pool. Cells were mixed with glycerol 50% in a 1:1 ratio and stored in 1 ml aliquots in cryotubes at −80°C.

To obtain a phage resistance mutant pool, a Tn-seq mutant library glycerol stock was used to inoculate 25 ml of LB supplemented with Cm and Km and incubated for 16 h at 30°C with shaking (160 rpm). The culture was adjusted to an OD_600_= 0.05, divided into two 5 ml flasks and infected with moi = 0 (uninfected control) and moi = 1 (challenged sample) respectively and incubated for 8 h at 30°C with shaking (160 rpm). Cells were collected by centrifugation at 4000 *g* for 15 min at 4°C. Following collection the pelleted cells were washed twice with phosphate buffered saline (PBS) to remove excess phage. After each wash the cells were pelleted by centrifugation. Finally, the cells were frozen at −20°C. The experiment was repeated in biological triplicates.

### DNA extraction and sequencing of transposon mutant libraries

For genomic DNA (gDNA) extraction the challenged pool samples were resuspended in 200 μl of LB and the uninfected controls were further diluted (1:10) due to high OD. Genomic DNA was extracted from cells using the DNeasy tissue kit (QIAGEN), following the manufacturer's instructions. Sequencing libraries were constructed as described elsewhere (35,36) using the NEBNext Ultra II FS DNA library Prep Kit for Illumina. Briefly, two rounds of PCR enrichment were used. In round one, primers PF3140 that binds to the adaptor and PF3139, a biotinylated primer that binds within the transposon were used. All primers are listed in Supplementary Table S3. Biotinylated PCR products were captured with Dynabeads M-270 Streptavidin (Invitrogen) following the manufacturer's instructions. The beads were used as template in the second PCR round with a nested Tn primer (PF3270) and a unique indexing primer (NEBNext Multiplex Oligos for Illumina). Library quality was assessed on an Agilent Bioanalyzer 2100 using a High Sensitivity DNA Kit. Libraries were further assessed through quantitative PCR (KAPA Library Quantification Kit, Universal, catalogue no. KK4824) using primers PF3124–PF3125 to determine molarity of fragments with Illumina P5/P7 ends (sequences required for flow-cell hybridization). Tn sequencing primer PF2926, along with PF3125, were used to determine the percentage of fragments containing true Tn sequences. Libraries were quantified using a Qubit fluorimeter and dsDNA HS Kit (Thermo Fisher Scientific) and diluted to 10 nM based on Qubit concentration and average fragment size. Libraries were then pooled with 10% PhiX control library and loaded at 1.5 pM (for low diversity) using a MiSeq Reagent Kit v.3 150 cycle kit for Illumina. Libraries were sequenced for 75 cycles (single ended) using custom sequencing primer PF2926 and Illumina Read 1 primer PF3441 (for PhiX library) at the Otago Genomics Facility (OGF). Sequencing with PF2926 generates a 12-nt transposon ‘tag’ to identify reads originating from Tn junctions.

### Tn-seq data analysis

Samples were de-multiplexed based on index sequence using standard Illumina software. FASTQ files were trimmed from the 3′-end to 50 nt using PRINSEQ lite and mapped to reference sequences using the Bio-Tradis pipeline (https://github.com/sanger-pathogens/Bio-Tradis) (37). The following parameters were used to run the bacteria_tradis script, which identifies the transposon tag in FASTQ files, then maps reads to the reference genome: ‘–smalt –smalt_k 10 –smalt_s 1 –smalt_y 0.92 -mm 2 -v -f filelist.txt -t TATAAGAGACAG -r laca.fasta’, where -smalt specifies the SMALT read mapping tool (https://www.sanger.ac.uk/tool/smalt/), -smalt_k specifies kmer size for reference fasta, -smalt_s specifies step size for kmers, -smalt_y specifies minimum percent identity of identical bases between read/reference, -mm specifies mismatches allowed in transposon tag sequence, -smalt_r specifies that reads that map equally in multiple places are discarded, -t specifies the transposon tag sequence, -r specifies the reference sequence, and -f indicates the list of FASTQ files to be processed. In summary, only reads containing the Tn tag (minimum 10/12 identity) and 92% identity to the reference sequence were considered for further analysis. Plot files (.insert_site_plot), which tabulate the number of reads at each nucleotide position in the reference sequence (plus and minus strands), were generated by the bacteria_tradis script. Subsequent analysis was performed in R using customized scripts based on http://doi.org/10.5281/zenodo.4554398. Plot files were used to generate a table of unique Tn insertion sites (for each sample). Feature enrichment was determined using differential expression analysis (exact methods—classic) of unique insertions in edgeR (38). The uninfected samples served as the control group, against which the phage challenged samples were compared. We then considered features with an adjusted *P*-value (*P*_adj_)<0.05 (Benjamini–Hochberg false discovery rate (FDR) correction) and a log_2_ (fold-change (FC)) >0.5 as host factors necessary for jumbo phage infection. The first replicate (sample 1 for both uninfected and infected) had fewer total transposon insertions and lower transposon insertion densities compared with the other two replicates (Supplementary Table S4, Supplementary Figure S1).

### Generation of knockdown strains by CRISPRi

To validate the Tn-seq results, knockdown strains of the candidate genes were generated using CRISPRi. Guide RNAs (gRNAs) were designed to target the non-template strand of the upstream region of candidate genes adjacent to the protospacer adjacent motif (PAM) (5′-NGG-3′) and introduced into a sniper dCas9 HF expression vector (pPF1755). dCas9 binding to the non-template strand has been shown to have a stronger repressive effect via CRISPRi than targeting the template strand (39,40). Hence, sgRNAs were designed to target the non-template strand of the first gene in the operon that was significantly enriched in the phage challenged samples. Inserts carrying the gRNA sequences were designed in overlapping oligos (forward and reverse primers, Supplementary Table S3) flanked by sequences compatible with BsaI overhangs. Oligos were annealed and introduced into a dCas9 expression vector, pPF1755 using a variant of Golden Gate cloning (41), transformed into *E. coli* ST18 heat shock competent cells and plated onto LB supplemented with Km and ALA (Supplementary Table S2 & Supplementary Table S7). Correct cloning of each gRNA sequence was confirmed by PCR using screening primers PF5508 and PF5509 and Sanger sequencing (Macrogen, South Korea). Finally, dCas9 constructs were conjugated into *Serratia*. In brief, the conjugations were carried out as follows: cultures of *E. coli* ST18 containing a dCas9 construct were washed with PBS to remove media containing Km. Each washed culture of *E. coli* was mixed with an overnight culture of *Serratia* sp. ATCC 39006 LacA and plated as spots onto LB agar plates containing no antibiotics. The conjugation spots were re-streaked twice onto LB agar supplemented with Km.

### Efficiency of plaquing assays

To assess the infectivity of *Serratia* phages (Supplementary Table S1) on CRISPRi knockdown strains, efficiency of plaquing (EOP) assays were performed. Overnight cultures of *Serratia* knockdown strains containing dCas9 expression plasmids with gRNA inserts were grown in LB supplemented with Km and 0.1% w/v arabinose at 30°C with shaking (120 rpm). A molten LBA overlay (4 ml, 0.35% w/v) previously seeded with 100 μl of bacterial culture was poured onto LBA plates supplemented with Km and arabinose (0.1% w/v). Serial tenfold dilutions of high-titre phage stock (∼10^11^ PFU/ml) were spotted (4–5 μl) onto the agar overlay and plates were incubated overnight at 30°C. The EOP was calculated as the ratio of PFU/ml produced on CRISPRi strains and the PFU/ml on the untargeted control (EV: empty vector, pPF1755). All conditions were repeated in three biological replicates and plotted as the mean ± SD.

### Infection in liquid culture

*Serratia* knockdown strains were grown overnight in LB with Km and arabinose and used to start fresh cultures (180 μl) from an OD_600_= 0.05 in 96-well plates. Strains were infected with PCH45 at moi = 0.01 by adding 20 μl of diluted phage lysate or phage buffer for the uninfected control. The 96-well plates were incubated in the plate reader (Clariostar) at 30°C with shaking (200 rpm) and bacterial growth was monitored by measuring OD_600_ every 12 min for 20 h. The experiment was repeated in biological triplicates (EV = 21, 3 per run). For each knockdown strain, bacterial growth and phage resistance were determined using the area under the growth curve (AUC) after 12 h incubation. Bacterial growth was calculated as the AUC of strains without phage infection, relative to the untargeted control (EV, pPF1755). Phage resistance was calculated as the AUC during infection (moi = 0.01) compared to the AUC when no phage was added and was then adjusted to EV (‘no resistance’) = 0% by subtracting the resistance value of EV, and *flhD* (‘full resistance’) = 100% by normalising the data to the resistance value of *flhD* and converting to percentage (%).

### Adsorption assay

*Serratia* knockdown strains grown overnight in LB (supplemented with Km + 0.1% w/v arabinose) were used to start fresh cultures (100 μl overnight culture + 900 μl LB) that were grown for 3 h and then adjusted to OD_600_= 0.3. Strains were infected with PCH45 at moi = 0.1 by adding an appropriate dilution of phage lysate or phage buffer to the uninfected control, and were incubated for 10 min. The incubated strains were centrifuged at 3220 *g* at 4°C for 5 min and the supernatant was collected and treated with NaHCO_3_ saturated chloroform. Serial tenfold dilution series of each supernatant were spotted (5 μl) onto an agar overlay containing *Serratia* and incubated overnight at 30°C. Adsorption was plotted as free phage (PFU/ml) remaining in supernatant for each infected strains and the phage containing bacteria-free control. All conditions were repeated in three biological replicates and plotted as the mean ± SD.

### Swimming assay

Overnight cultures of *Serratia* knockdown strains were grown in LB supplemented with Km and arabinose (0.1% w/v) at 30°C with shaking (120 rpm). Sterile toothpicks were submerged in each overnight culture, stabbed into 0.3% tryptone swarm agar (TSA; 10 g tryptone, 5 g NaCl, 3 g agar, 1 L distilled water) with Km and 0.1% w/v arabinose and incubated at 30°C for ∼20 h. Swimming ability was determined by measuring the diameter of the swimming halo in comparison to an untargeted control. The experiment was repeated in biological triplicates (EV = 9 biological replicates, 3 per TSA plate).

## Results

### Genome-wide screening identifies host factors involved in nucleus-forming jumbo phage infection

**Figure 1.**
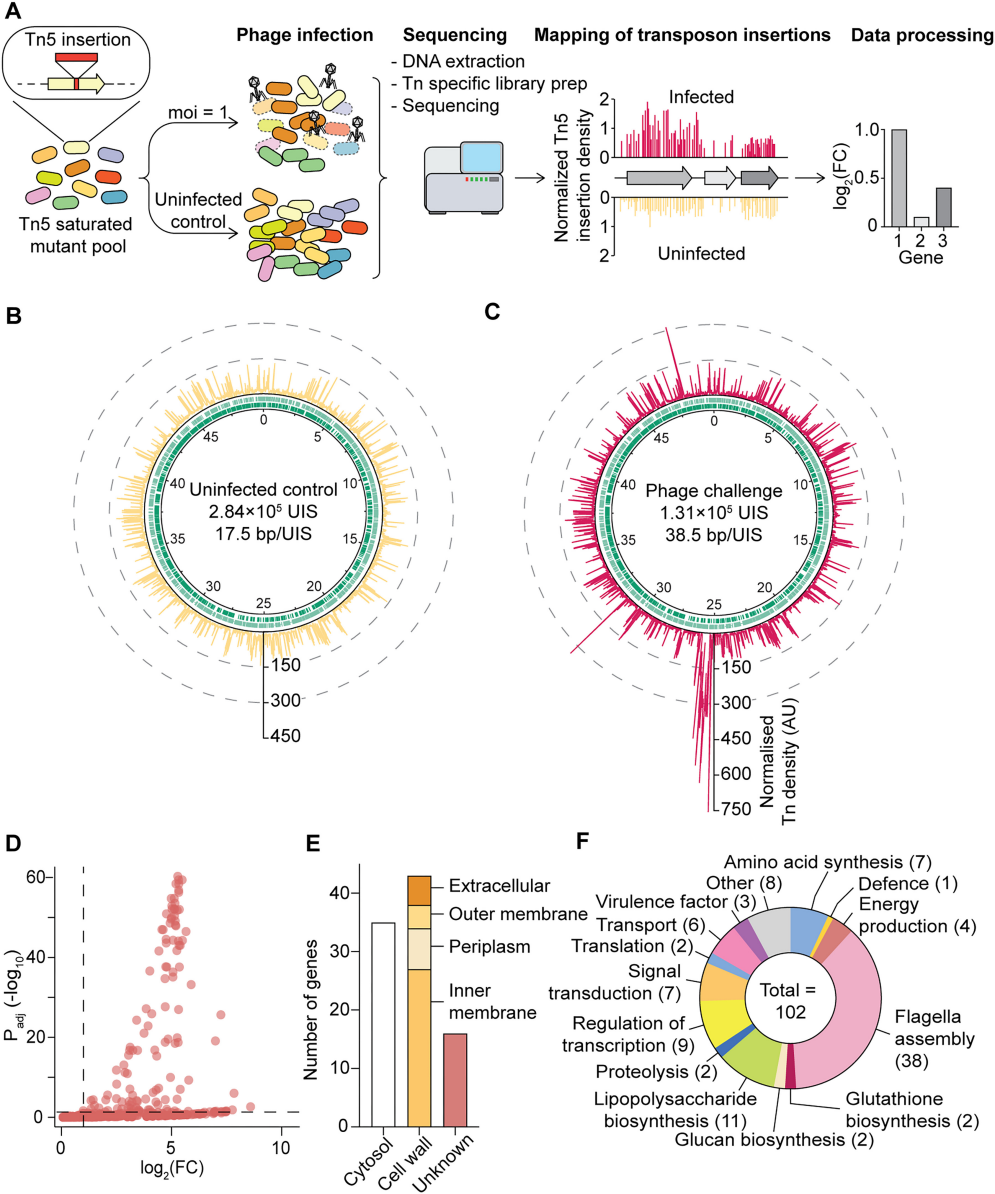
Bacterial genes involved in diverse bacterial processes are involved in nucleus-forming phage infection (**A**) Overview of Tn-seq approach. Transposon insertion density across the *Serratia* genome with (**B**) uninfected samples and (**C**) phage challenged samples. Inner circles represent genes encoded in the forward (light grey) and reverse strand (dark grey). UIS: Unique insertion sites. Each insertion was normalised to the total number of unique insertions in the sample (normalised Tn density, AU (arbitrary unit)). (**D**) Volcano plot of non-essential *Serratia* genes significantly enriched during phage infection (log_2_(FC) > 0.5 and *P*_adj_< 0.05). (**E**) Predicted location of proteins in the cell. (**F**) Tn-seq hits were classified into functional groups through gene ontology (GO) (42,43). Some genes were classified into more than one functional group.

To determine bacterial genes contributing to infection by a nucleus-forming jumbo phage (PCH45), Tn-seq was employed (Figure 1A). First, we generated a *Serratia* transposon-saturated mutant library (Tn*5*-derivative) and infected it with PCH45. After 8 h of infection, we performed high-throughput transposon insertion-site sequencing, and mapped transposons to the bacterial genome in phage-infected samples and the uninfected controls. After infection, bacteria with transposon-inactivated genes that contribute to phage infection will be enriched in the challenged samples (i.e. their mutation results in phage resistance or reduced infection) provided the gene is non-essential for the bacterial host (Figure 1A). We performed three paired replicates of both the uninfected control and phage infected experiments. Our sequencing depth was sufficient to detect most mutants present in each sample (Supplementary Figure S1A). Combined, we observed 353 093 unique Tn insertions across the six conditions (3× uninfected and 3× infected) (Supplementary Table S4). The three uninfected samples had a total of 283 677 unique insertions (17.5 bp/UIS (unique insertion sites)), while phage challenged samples harboured a total of 130 619 unique insertions across the 3 replicates (38.5 bp/UIS) (Figure 1B, C and Supplementary Table S4). By comparing the transposon density across the *Serratia* genome without or with phage infection, we identified genomic regions enriched with transposons after phage challenge. Our analysis showed that 94 genes and 34 intergenic regions were significantly enriched (log_2_(FC) > 0.5 and *P*_adj_< 0.05) after PCH45 infection (Figure 1D, Supplementary Table S5 and Supplementary Table SE1).

To understand which processes were involved in nucleus-forming phage infection we examined the genomic context of mutant loci enriched upon phage challenge. Most genes significantly enriched in the phage challenged sample were grouped in operons (60 genes). In total, 40 operons were identified that contained at least one (22 operons) or two (18 operons) significantly enriched genes. The remaining 12 genes that were enriched were encoded separately (standalone). More than 80% of the enriched intergenic regions (28 out of 34 intergenic regions) were located upstream of genes also found in our analysis, or as part of the enriched operons (Supplementary Table S6).

Next we classified the enriched loci by cellular localisation and predicted function. Forty three host factors were predicted to be located at the cell wall—in the inner membrane (27 proteins), periplasm (7 proteins), outer membrane (4 proteins) or extracellular (5 proteins). In contrast, 35 proteins were predicted to be cytoplasmic and the location of 16 were unknown (Figure 1E, Supplementary Table S7). Next, we predicted function based on gene ontology (Figure 1F, Supplementary Table S7) (42,43). Almost half of the enriched genes were involved in flagella (38 genes, ∼35%) and lipopolysaccharide (11 genes, ∼11%) biosynthesis. Other genes enriched were involved in transcriptional regulation (9 genes), signal transduction (7 genes), energy production (4 genes), amino acid biosynthesis (7 genes), translation and ribosomal biosynthesis (2 genes), and transport (3 genes). Our results demonstrate the suitability of Tn-seq as a genome-wide approach to identify host loci influencing nucleus-forming phage infection and to rapidly assess mutations that can cause phage resistance. Altogether, we uncovered 94 bacterial genes involved in diverse processes necessary for nucleus-forming phage infection.

### Mutations in flagella biosynthesis genes are a major cause of phage resistance

**Figure 2.**
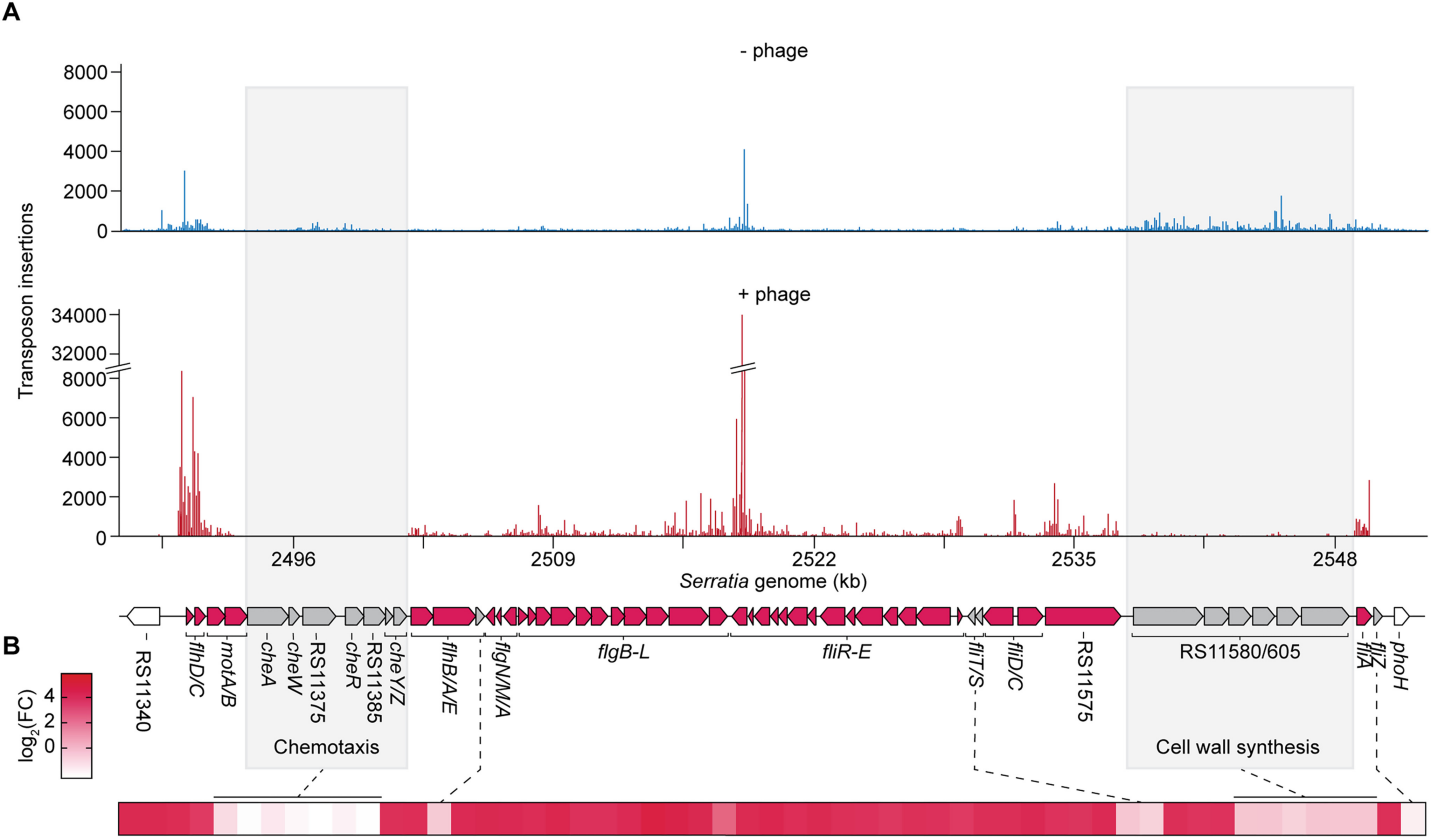
Mutation of flagella assembly and biosynthesis genes is a common route to jumbo phage resistance. (**A**) Transposon insertion density across the flagella locus in *Serratia* and schematic of the flagella gene cluster. Genes significantly enriched in the challenged sample (log_2_(FC) > 0.5 and *P*_adj_< 0.05, pink), genes that did not reach significance (grey). (**B**) Heat map of log_2_(FC) in flagella gene cluster.

Our screen identified many genes encoding surface-associated properties. In the adsorption step, phages recognise specific cell-surface receptors (44,45) and PCH45 requires flagella to infect *Serratia* (36). In agreement, one third of the genes significantly enriched in the phage challenged sample (∼35%, 38 genes) were involved in flagella biosynthesis (Figure 1F). In *Serratia*, flagella biosynthesis involves 55 genes clustered together in multiple operons (Figure 2A). Most genes in the flagella gene cluster, including genes involved in flagella rotation (such as *motA* and *motB*), biosynthesis and structural genes (*flhAB*, *flgA-N* and *fliA-R*), and regulation (flagella master regulator *flhDC*, RNA polymerase sigma factor *fliA* and anti-sigma factor *flgM*) showed an increased transposon insertion density in the phage challenged sample (Figure 2A, B). These results suggest that complete and rotating flagella are required for PCH45 phage infection. Interestingly, genes encoded in the flagella gene cluster involved in chemotaxis (*cheABWRYZ* and the HAMP domain-containing gene RS11375), and cell wall formation (RS11580 to RS11605) were not necessary for nucleus-forming phage infection. Our results suggest that mutations that prevent phage adsorption, in this case through mutation of flagella, is an efficient way to prevent PCH45 phage infection.

### CRISPRi silencing of the flagella master regulator and the Rcs pathway elicits jumbo phage resistance

**Figure 3.**
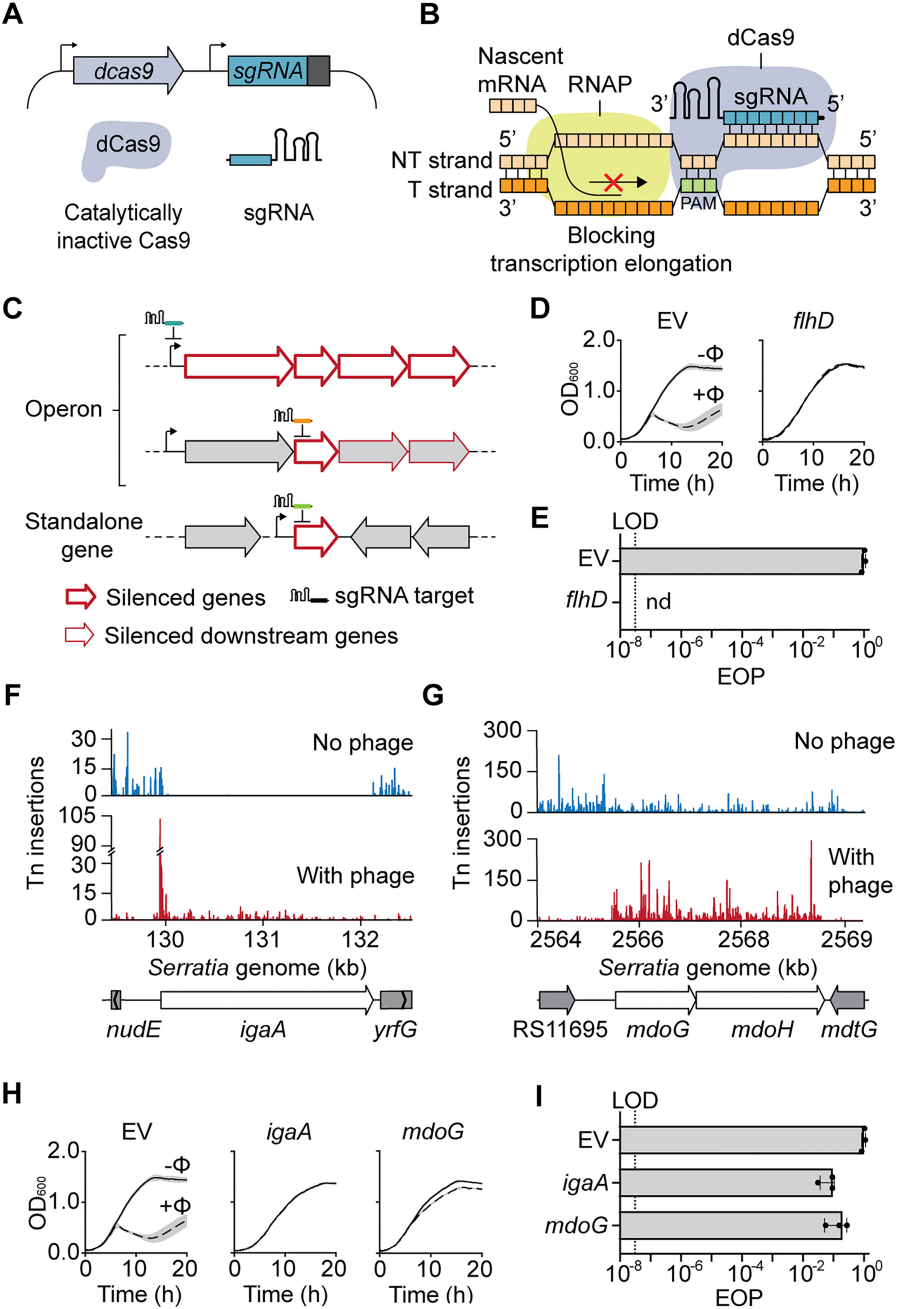
CRISPRi enables validation of gene candidates involved in nucleus-forming phage infection. Silencing of host genes necessary for nucleus-forming phage development was performed through CRISPRi. (**A**) Both dead Cas9 (dCas9) and a sgRNA were expressed from a plasmid. (**B**) Single guide RNAs (sgRNAs) were designed to target the gene of interest and expressed alongside a nuclease dCas9 in *Serratia*. Binding of dCas9-sgRNA to the target hampers transcription by blocking RNA polymerase. (**C**) Single guide RNA (sgRNA) design. (Upper) sgRNA were designed to target the start of an operon when all or most of the genes encoded downstream were enriched in the Tn-seq analysis. (Middle) sgRNAs were designed to target the non-template strand of the first gene in the operon that was significantly enriched in the phage challenged samples. (Lower) For standalone genes, guides targeting the start of the gene were designed. (**D**) Infection in liquid culture upon PCH45 infection of *Serratia* containing empty vector (EV, pPF1755) and *flhD* knockdown strain and (**E**) efficiency of plaquing (EOP). LOD = limit of detection and nd = none detected. Transposon insertion density in the (**F**) *igaA* and (**G**) *mdoGH* locus. (**H**) Bacterial growth in liquid culture upon PCH45 phage infection of *Serratia* containing EV, *igaA* and *mdoG* knockdown strains and (**I**) EOP. Data in (D), (E), (H) and (I) are represented as the mean of biological triplicates ± SD.

The Tn-seq method enabled a highly-parallel, genome-wide, mixed population survey of which genes influence phage infection. To further understand the involvement of these genes individually during nucleus-forming phage infection, we used CRISPR interference (CRISPRi) transcriptional knock-down constructs. CRISPRi uses a nuclease-deficient variant of Cas9 (dCas9), paired with a single guide RNA (sgRNA) with complementarity to the promoter or coding region of a gene of interest (Figure 3A) (46). Target binding by dCas9 disrupts RNA polymerase binding or advancement and hinders transcription elongation (Figure 3B) (47). Therefore, CRISPRi represses the transcription of genes within an operon that are encoded downstream of the target gene (47). Since our results show that most of the genes significantly enriched by Tn-seq were in operons (60 out of 94, 63%); we took advantage of the polar effects of CRISPRi and designed sgRNAs targeting the first gene in each operon that was significantly enriched in our phage challenged samples (Figure 3C). To first test the efficacy of CRISPRi, we targeted the flagella master regulator *flhD* and performed phage infection assays on the knockdown strain. In agreement with our Tn-seq results, the knockdown of *flhD* led to undetectable phage infection on solid and liquid media (Figure 3D, E).

Previously, activation of the Rcs phosphorelay was shown to provide protection against PCH45, through downregulation of flagella (36). In agreement, three genes involved in the induction of the Rcs signaling pathway (when mutated), *igaA*, *mdoG* and *mdoH*, were enriched by Tn-seq upon phage challenge (Figure 3F, G). We generated CRISPRi knockdown strains with sgRNAs targeting *igaA* or the *mdoGH* operon, which both showed increased resistance to phage PCH45 in liquid and solid media infection assays (Figure 3H, I, Supplementary Table SE2 and Supplementary Table SE3). In summary, CRISPRi enabled the validation of host genes from our Tn-seq screening, such as *flhD*, *igaA* and *mdoGH*, facilitating the investigation of their involvement in nucleus-forming phage infection.

### CRISPRi validates genes identified by Tn-seq involved in phage resistance

**Figure 4.**
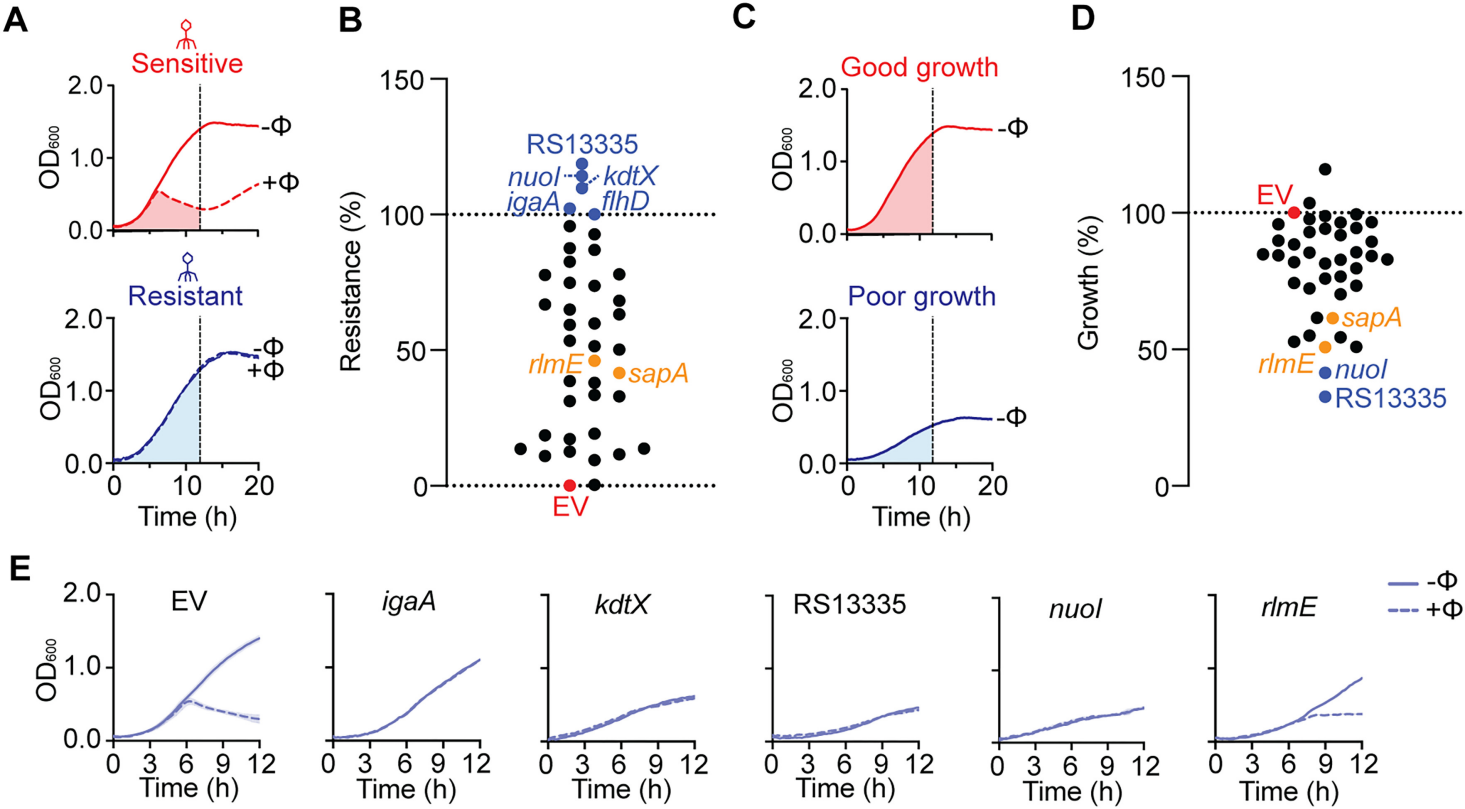
Jumbo phage infection in liquid culture validates Tn-seq results. (**A**) Example of jumbo phage infection in sensitive or resistant strains. Area under the curve was used to calculate phage resistance (i.e. area under the curve up to 12 h upon phage challenged compared with no phage). (**B**) Phage resistance of knockdown strains relative to EV (i.e. *Serratia* WT with pPF1775, example of ‘no resistance’ i.e. 0%) and *flhD* (example of ‘full resistance’ i.e. 100%), see methods. Blue dots indicate knockdown strains display phage resistance similar to the knockdown of the primary receptor (*flhD*). Orange dots indicate strains with resistance less than the *flhD* knockdown and with growth defects. (**C**) Example of growth phenotypes in liquid growth curve assays. Area under the curve used to calculate bacterial growth is shaded in both instances (see methods). (**D**) Bacterial growth of knockdown strains relative to EV. (**E**) Liquid growth curves of knockdown strains demonstrating phage resistance similar to the primary receptor knockdown strain (*flhD*), except *rlmE* which provides an example of a knockdown strain with poor growth displaying moderate phage resistance. Infection in liquid culture upon PCH45 phage infection of *Serratia* knockdown strains is represented as the mean of biological triplicates ± SD.

To examine the role of the other genes identified by Tn-seq in nucleus-forming phage infection, we used CRISPRi silencing in phage assays. To assess phage resistance of each knockdown strain we examined bacterial growth in liquid, which mimicked the Tn-seq conditions (see Supplementary Figure S2, Supplementary Table SE2 and Supplementary Table SE3 for liquid growth curves for all knockdown strains). Phage resistance of each knockdown strain was calculated as a ratio of phage infection relative to a sensitive empty vector control strain (see methods and Figure 4A). All knockdown strains showed phage resistance levels equal to or higher than the empty vector control (Figure 4B). Of all the knockdown strains assessed, four (RS13335, *nuoI*, RS01810, *igaA*) showed similar levels of resistance to the flagella regulator (*flhD*) knockdown, indicating these genes are vital to enable PCH45 infection (Figure 4B).

We observed that the knockdown of some genes resulted in impaired bacterial growth. To compare growth defects among knockdown strains we calculated a ratio of growth relative to our empty vector strain (see methods) and also visualised impairments in growth curve assay plots (Figure 4C–E, Supplementary Figure S2 and Supplementary Table SE2 and Supplementary Table SE3). Three knockdowns (*nuoI*, *rlmE* and *RS13335*) impaired growth defects by approximately 50% (Figure 4D, E). Interestingly, knockdown of two of these genes, encoding the NADH-quinone oxidoreductase subunit NuoI and hypothetical protein RS13335, also elicited high phage resistance (Figure 4B, E). It is feasible that lower metabolic activity and growth may limit phage infection and contribute towards resistance. However, bacterial growth does not always correspond to the strength of phage resistance. For example, the knockdown of *sapA* and *rlmE* leads to only moderate levels of phage resistance but causes a major growth defect (Figure 4B, D). Overall, CRISPRi confirmed that most host factors identified in a pooled approach by Tn-seq are required for successful nucleus-forming phage infection when examined individually.

### Silencing non-flagellar genes that influence bacterial motility provides phage resistance

**Figure 5.**
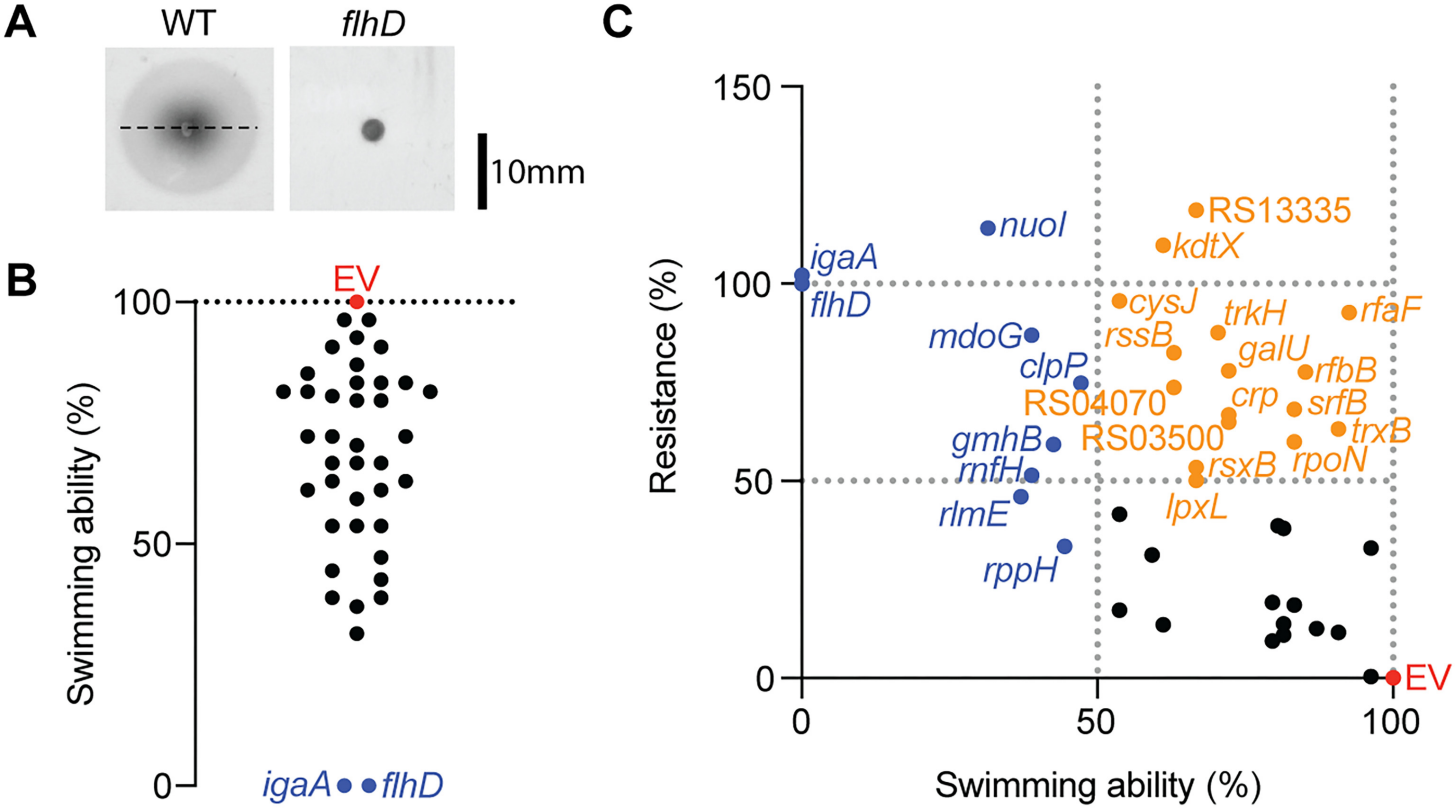
Silencing of non-flagellar genes involved in bacterial motility provide phage resistance. (**A**) Example of swimming assay. Swimming ability (%) was calculated as the diameter of the swimming halo of each knockdown strain in relation to the swimming halo of the empty vector (EV, pPF1755) strain. (**B**) Swimming (%) of knockdown strains (mean of three biological replicates) relative to EV. (**C**) Swimming compared with phage resistance of knockdown strains. Blue dots indicate strains with less than 50% swimming ability. Orange dots indicate strains with >50% swimming ability and >50% resistance.

Due to the importance of flagella-mediated motility, diverse genes indirectly influence flagella biosynthesis and motility (48) and therefore, may affect phage resistance. To test this, we performed swimming assays on all knockdown strains (Figure 5A, B, see all CRISPRi-based knockdown strain swimming abilities in Supplementary Figure S3 and Supplementary Table SE2). This confirmed that motility was decreased in *flhDC*, *igaA* and *mdoGH*, which were known to downregulate flagella (36). Knockdowns of other genes involved in various cellular functions (*nuoI*, *clpP*, *rlmE*, *gmhB*, *rnfH*, RS19675) led to strongly impaired motility (<50%). For example, knockdowns of operons involved in energy production (*nuoI*), translation (*rlmE*), and proteolysis (*clpP*) showed decreased swimming and displayed phage resistance (Figure 5B, C). The NADH-ubiquinone oxidoreductase, encoded by *nuoA-N*, forms respiratory complexes that produce the proton motive force that helps drive flagella rotation (49). Interactions between the flagellar switch protein (FliM) and NuoC (NADH dehydrogenase I) in both *Campylobacter jejuni* and *Escherichia coli* suggest this respiratory complex could also be directly involved in motility through posttranslational regulation (50). Our screen also highlighted 16 genes with moderate to high (>50%) swimming ability paired with a moderate to high (>50%) resistance profile, with the majority being involved in lipopolysaccharide synthesis (*rfbB, galU, rfaF, kdtX, kdsD, lpxL*), and others involved in the regulation of transcription (e.g. *rpoN*), virulence (e.g. *srfB*) and transport (e.g. *trkH*). Our results show that disruption of many cellular pathways can impact flagella-based motility thus affecting nucleus-forming phage infection, however there are multiple host factors that have lesser effects on flagella that also confer phage resistance.

### LPS genes are important for PCH45 infection

**Figure 6.**
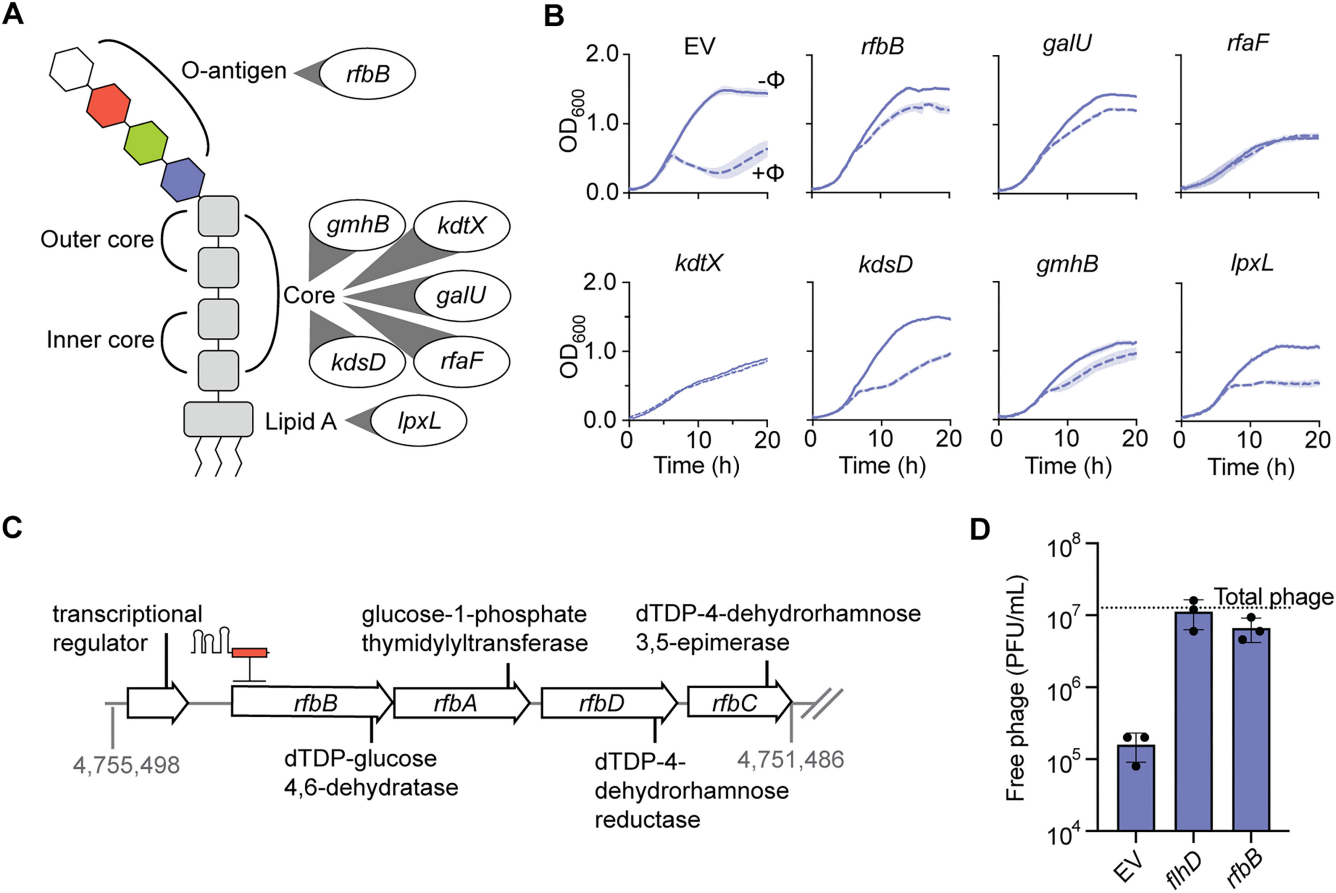
Changes to the LPS biosynthesis impact jumbo phage infection. (**A**) Schematic of an LPS molecule with associated host factor locations from LPS biosynthetic knockdown strains. (**B**) Phage resistance effects of CRISPRi-based knockdowns of different LPS biosynthesis genes assessed in bacterial growth assays. Infection in liquid culture upon PCH45 phage infection of *Serratia* WT is represented as the mean of biological triplicates ± SD. (**C**) Schematic of the *rfbBADC* operon. (**D**) Jumbo phage adsorption plotted as free phage in PFU/mL unabsorbed by each knockdown strain tested. Free phage (PFU/mL) is represented as the mean of biological triplicates ± SD. Total phage represents the total amount of phage added at the beginning of infection.

The outer membrane of gram-negative bacteria, such as *Serratia*, contains lipopolysaccharides (LPS) that consist of a hydrophobic lipid domain (lipid A), a core region, and an O-antigen region containing variable oligosaccharides (51) (Figure 6A). Having identified 11 genes associated with LPS biosynthesis enriched in the phage challenged sample, we hypothesized that PCH45 employs both flagella and LPS for adsorption. The genes identified encode components for the biosynthesis of the core region (*kdsD*, *kdtX*, *rfaF*, *galU* and *gmhB*) O-antigen (*rfbBADC*) and lipid A (*lpxL*) (Supplementary Table S5). Knockdowns of each LPS strain led to phage protection, with some (*rfbB*, *galU*, *rfaF* and *gmhB*) having strong phage resistant phenotypes (Figure 6B, Supplementary Figure S2, Supplementary Table SE2 & Supplementary Table SE3). Of these knockdown strains, all except *gmhB* displayed minimal swimming impairments, suggesting these LPS biosynthetic genes could be acting as a secondary receptor in nucleus-forming phage infection (Figure 5C, Supplementary Figure S3, Supplementary Table SE2). Next, we tested the effect of silencing the *rfbBADC* operon predicted to be involved in O-antigen modification (dTDP-rhamnose) to see if phage adsorption was affected by minor changes to the LPS (Figure 6C, D). The knockdown of genes predicted to be involved in dTDP-rhamnose production led to a large decrease in PCH45 adsorption (Figure 6D, Supplementary Table SE2), suggesting minor changes to LPS directly effects adsorption. Overall, jumbo phage PCH45 may use an adsorption strategy involving LPS, alongside its main receptor, flagella.

### Nucleus-forming jumbo phages require cellular pathways also used by other phages

**Figure 7.**
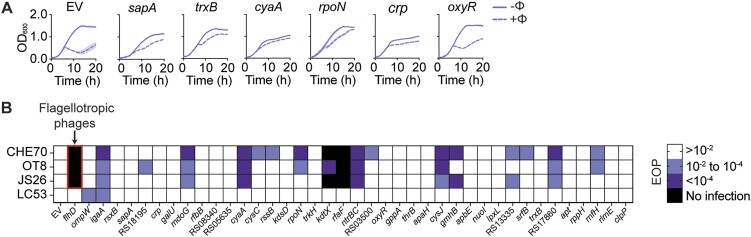
Flagellotropic phages infecting *Serratia* use similar host factors to the nucleus-forming phage PCH45. (**A**) Liquid growth curves of knockdown strains of the *sapABCDF* operon, *trxB* and transcriptional regulator genes *cyaA*, *rpoN*, *crp* and *oxyR*. Infection in liquid culture upon PCH45 phage infection of *Serratia* containing EV (pPF1755) is represented as the mean of biological triplicates ± SD. (**B**) Heatmap of CRISPRi strains against a panel of *Serratia* phages; flagellotropic jumbo: CHE70, flagellotropic non-jumbo: JS26, OT8 and a non-flagellotropic non-jumbo control phage: LC53. Colours correspond to groups with a fold decrease in infection of: >10^−2^ (white), 10^−2^–10^−4^ (light purple), <10^−4^ (dark purple) and no infection (i.e. none detected) (black) (adjusted to EV, pPF1755) derived from EOP assay data.

It has been proposed that jumbo phages act more independently of their host cells compared with non-jumbo phages due to their large accessory genome (7). However, in our study we found that nucleus-forming phages employ many host genes also reported to be involved in non-jumbo phage infection. For example, the susceptibility to antimicrobial peptides (*sapABCDF*) operon, alongside the potassium uptake system TrkH, which are both implicated in T4 phage infection (52) and *Salmonella* phages Aji_GE and FelixO1 (53) were enriched in our Tn-seq screen and had phage resistant phenotypes in liquid growth conditions (Figure 7A, Supplementary Table S5, Supplementary Figure S2 and Supplementary Table SE2 and Supplementary Table SE3). Interestingly, *sapD* and *sapF* were the two most strongly enriched genes, indicating they are vital to PCH45 phage infection (log_2_(FC)=7.76 and 7.84 respectively) (Supplementary Table S5). Additionally, thioredoxin-disulfide reductase *trxB*, which has been implicated in phage T7 DNA polymerase activity and filamentous phage infection (54,55), was also involved in nucleus-forming jumbo phage infection (Figure 7A, Supplementary Figure S2, Supplementary Table S5 and Supplementary Table SE2 and Supplementary Table SE3). Other Tn-seq based approaches have reported results consistent with our findings. For example, intracellular growth attenuator (*igaA*), the RNA polymerase sigma-54 factor (*rpoN*), DNA-binding transcriptional regulator CRP (*crp*), adenylate cyclase (*cyaA*) and hydrogen peroxide-inducible genes activator (*oxyR*) were also involved in non-jumbo phage infections (53,56–59) (Figure 7A, Supplementary Figure S2, Supplementary Table S5 and Supplementary Table SE2 and Supplementary Table SE3).

To experimentally test the specificity of these bacterial genes to nucleus-forming jumbo-phages, we used our CRISPRi knockdown panel to compare infection by different *Serratia* phages. Three flagella-dependent phages were tested: JS26 (Siphovirus), OT8 (Siphovirus) and CHE70 (predicted non-nucleus forming jumbo phage, 265 422 kb). We also included LC53, which is a non-jumbo phage of the Winklervirus family related to T4 that uses OmpW as a receptor (33). We demonstrated that flagellotropic *Serratia* phages (both non-jumbo and jumbo) require some of the host genes used by the nucleus-forming phage PCH45 (Figure 7B, Supplementary Table SE2). Notably, the jumbo phage CHE70, shares the highest amount (*n*= 17) of the host genes used by jumbo phage PCH45. Interestingly, the T4 related phage LC53, was only impaired by an *igaA* knockdown, or when the *ompW* receptor expression was silenced. In agreement, we previously demonstrated that deletion of *igaA* led to a decrease in *ompW* expression (36). These results demonstrate that under these *in vitro* conditions the receptor usage, rather than the genomic composition of the phage, is a major contributor that determines which bacterial mutations will result in phage resistance.

## Discussion

Recently, there has been major interest in nucleus-forming phages, due to their complex developmental process involving nucleus formation, their evasion from DNA-targeting anti-phage defences and their potential in phage therapies. We set out to combine a genome-wide strategy, Tn-seq, with a precise genetic knockdown approach, CRISPRi, to identify bacterial genes influencing nucleus-forming jumbo phage infection. The aim was to reveal which non-essential genes are involved in nucleus-forming phage infection and in turn uncover how resistance to nucleus-forming phage infection could emerge by gene inactivation. In total, we identified 94 non-essential genes associated with *Serratia* nucleus-forming jumbo phage PCH45 infection.

Nucleus-forming phages, with their ability to shield their genome from DNA-targeting defence systems, can function as antimicrobials against multidrug resistant (MDR) bacteria that encode many phage defence systems (15,16). Our major finding was that most mutations impacting phage infection were in phage receptors (flagella and LPS) or intracellular genes that influence receptor expression or function. These results are consistent with other Tn-seq studies, which have identified receptors for multiple phages, including nucleus-forming jumbo phage φKZ (53,56,57,60,61). Recently, the *Klebsiella*-infecting flagellotropic phage fENko-Kae01 was isolated, where phage resistance arose through flagella and Rcs pathway mutations (62). Therefore, receptor inactivation is an efficient way to escape phage infection – at least under laboratory conditions. Indeed, it is well understood that bacteria can evade phage attachment by either mutating, or reducing the expression of, receptors (36,63). Likewise, L-form (cell wall deficient) bacteria lack receptors and are phage resistant (64). However, receptor mutations often have a fitness cost, which can be disadvantageous in non-clonal populations of bacteria, with other phage resistance mechanisms, such as CRISPR-Cas, favored (65,66). Our Tn-seq protocol used a clonal population of *Serratia* paired with a high phage challenge, which would likely favor receptor mutations. Whilst we identified a large portion of genes contributing to PCH45 infection being involved in flagella (∼35%) or other genes contributing to flagella, we also discerned genes that did not affect phage receptor function, which revealed intracellular pathways that, when disrupted, can lead to nucleus-forming phage resistance. In a more complex environment where other bacteria are present, such as in a clinical setting, we may see a shift in the frequency of receptor mutations to intracellular resistance mechanisms.

Although nucleus-forming jumbo phages are considered to use less host factors for replication than non-jumbo phages (7), we demonstrated that many genes required for jumbo phage infection were also used by non-jumbo phages. Most importantly, the specific receptor utilized by the phage was the major contributor to the shared genetic requirements for infection which has also been observed in cross-resistance studies on *Pseudomonas*-phages (58). Therefore, nucleus-forming phage resistance provide cross resistance to other phages that use the same receptor. For phage therapy, our findings highlight the importance of selecting phages with distinct receptor profiles when designing phage cocktails (67), and that single jumbo phages will likely have similar therapeutic limitations as non-jumbo phages in terms of receptor-mediated resistance. However, phage resistance mutations can be exploited to ‘steer’ bacteria towards re-sensitization to antibiotics or reduce their virulence (67–69). Previous studies have shown that mutations (e.g. *igaA*) in the Rcs pathway result in virulence attenuation (69–72). Though we observed *igaA* provides pan-immunity to against many *Serratia* phages, it is unlikely an *igaA* mutation would occur in a clinical setting due to the loss of negative regulation of the Rcs pathway, virulence attention and resulting growth defects upon full inactivation (36,59,73,74). Collectively, the identification of the spectrum of conserved bacterial genes used by different phage families for infection may inform the selection of diverse phages for therapies that are less likely to yield cross-resistance. For example, we demonstrated that phage LC53 and PCH45 share distinct infection requirement and therefore would be a good pair to include in a cocktail. Identifying phage resistance pathways and genomic requirements for phage infection are paramount to develop successful phage-based therapies. To ensure the successful future use of phage cocktails, phages with diverse receptor requirements must be used to avoid the development of cross-resistance in MDR bacteria.

## Supplementary Material

gkae1194_Supplemental_Files

## Data Availability

Raw reads from Tn-seq analyses have been deposited at the NCBI Sequence Read Archive (SRA) under BioProject accession number PRJNA746616. Data analysis pipelines used for Tn-seq were https://github.com/sanger-pathogens/Bio-Tradis and http://doi.org/10.5281/zenodo.4554398. Reference sequences and annotations used in this study are available through NCBI: *Serratia* sp. ATCC 39006 LacA (CP025085.1 / assembly GCF_002847015.1_ASM284701v1).

## Notes

Present address: Lucia M. Malone, Brightlands Maastricht Health Campus, Maastricht, Netherlands.
