# Supplementary material for: Genome-wide identification of bacterial genes contributing to nucleus-forming jumbo phage infection
Source: Nucleic Acids Res. 2024 Dec 19;53(3):gkae1194. doi: 10.1093/nar/gkae1194 (PMC11797060; doi:10.1093/nar/gkae1194)
Supplement: gkae1194_Supplemental_Files [file gkae1194_supplemental_files.zip › Harding et. al. Supplementary Material.pdf]

Supplementary Material

Genome-wide identification of bacterial genes contributing to nucleus-forming jumbo phage infection

Kate R. Harding[1,2,4†], Lucia M. Malone[1,2,5†], Natalie A.P. Kyte[1], Simon A. Jackson[1-4], Leah M. Smith[1,2,4] and Peter C. Fineran[1-4*]

[1]Department of Microbiology and Immunology, University of Otago, PO Box 56, Dunedin 9054, New Zealand
[2]Genetics Otago, University of Otago, PO Box 56, Dunedin 9054, New Zealand
[3]Bioprotection Aotearoa, University of Otago, PO Box 56, Dunedin 9054, New Zealand
[4]Maurice Wilkins Centre for Molecular Biodiscovery, University of Otago, PO Box 56, Dunedin 9054, New Zealand
[5]Present address: Kavli Institute of Nanoscience, Delft University of Technology, Delft, Netherlands
[†]The first two authors contributed equally to this work.

[*]For correspondence: peter.fineran@otago.ac.nz

Supplementary Excel Files

Table SE1. Transposon sequencing edgeR output.

Table SE2. CRISPRi experimental data. Includes: efficiency of plating, swimming assay, growth curve assay and adsorption assay data.

Table SE3. CRISPRi growth curve data. Includes the targeting of each gene by two single guide RNAs for CRISPRi silencing.

Table S1. Bacterial strains and bacteriophages used in this study.

Strain	Description	Notes	Reference
Escherichia coli ST18	Auxotrophic donor for biparental conjugation	SmR, requires ALA	[1]
Serratia sp. ATCC 39006	lac EMS mutant, denoted WT		[2]
PCH45	*Serratia* phage		[3]
LC53	*Serratia* phage		[4]
OT8	*Serratia* phage		[5]
JS26	*Serratia* phage		[6]
CHE70	*Serratia* phage		This study

Table S2. Plasmids used in this study (CRISPRi constructs)

Name	Features	Description	Construction	Reference
pPF781	pBAD30, CmR, OriT, T4term, T7term	pBAD30 construct with CmR for counterselection against *E. coli* ST18 during transposon mutant pool generation		[7]
pPF1755	dCas9(HF1), KmR, OriT, RK2, AraC, PBAD	dCas9 construct with gRNA entre with BsaI cut sites		This study
pPF2685	dCas9(HF1), KmR, OriT, RK2, AraC, PBAD	dCas9 construct with gRNA-5 targeting *flhD*	pPF1755 (BsaI-HFv2) + PF5300/PF5267	This study
pPF2686	dCas9(HF1), KmR, OriT, RK2, AraC, PBAD	dCas9 construct with gRNA-6 targeting *flhD*	pPF1755 (BsaI-HFv2) + PF5268/PF5269	This study
pPF2687	dCas9(HF1), KmR, OriT, RK2, AraC, PBAD	dCas9 construct with gRNA-7 targeting *flhD*	pPF1755 (BsaI-HFv2) + PF5270/PF5271	This study
pPF2688	dCas9(HF1), KmR, OriT, RK2, AraC, PBAD	dCas9 construct with gRNA-8 targeting *flhD*	pPF1755 (BsaI-HFv2) + PF5272/PF5273	This study
pPF2689	dCas9(HF1), KmR, OriT, RK2, AraC, PBAD	dCas9 construct with gRNA-9 targeting *igaA*	pPF1755 (BsaI-HFv2) + PF5274/PF5275	This study
pPF2691	dCas9(HF1), KmR, OriT, RK2, AraC, PBAD	dCas9 construct with gRNA-11 targeting *rsxA* (note: *rsxB and rsxC* were the genes significantly enriched in this operon - this guide is labelled as '*rsxB*' in the manuscript)	pPF1755 (BsaI-HFv2) + PF5316/PF5317	This study
pPF2692	dCas9(HF1), KmR, OriT, RK2, AraC, PBAD	dCas9 construct with gRNA-12 targeting *rsxA* (note: *rsxB* and *rsxC* was the genes significantly enriched in this operon - this guide is labelled as '*rsxB*' in the manuscript)	pPF1755 (BsaI-HFv2) + PF5318/PF5319	This study
pPF2693	dCas9(HF1), KmR, OriT, RK2, AraC, PBAD	dCas9 construct with gRNA-15 targeting *sapA*	pPF1755 (BsaI-HFv2) + PF5320/PF5321	This study
pPF2694	dCas9(HF1), KmR, OriT, RK2, AraC, PBAD	dCas9 construct with gRNA-16 targeting *sapA*	pPF1755 (BsaI-HFv2) + PF5322/PF5323	This study
pPF2695	dCas9(HF1), KmR, OriT, RK2, AraC, PBAD	dCas9 construct with gRNA-21 targeting CWC46_RS18195	pPF1755 (BsaI-HFv2) + PF5324/PF5325	This study
pPF2696	dCas9(HF1), KmR, OriT, RK2, AraC, PBAD	dCas9 construct with gRNA-22 targeting CWC46_RS18195	pPF1755 (BsaI-HFv2) + PF5326/PF5327	This study
pPF2697	dCas9(HF1), KmR, OriT, RK2, AraC, PBAD	dCas9 construct with gRNA-23 targeting *crp*	pPF1755 (BsaI-HFv2) + PF5328/PF5329	This study
pPF2698	dCas9(HF1), KmR, OriT, RK2, AraC, PBAD	dCas9 construct with gRNA-24 targeting *crp*	pPF1755 (BsaI-HFv2) + PF5330/PF5331	This study

Name	Features	Description	Construction	Reference
pPF2699	dCas9(HF1), KmR, OriT, RK2, AraC, PBAD	dCas9 construct with gRNA-25 targeting *galU*	pPF1755 (BsaI-HFv2) + PF5332/PF5333	This study
pPF2700	dCas9(HF1), KmR, OriT, RK2, AraC, PBAD	dCas9 construct with gRNA-26 targeting *galU*	pPF1755 (BsaI-HFv2) + PF5334/PF5335	This study
pPF2701	dCas9(HF1), KmR, OriT, RK2, AraC, PBAD	dCas9 construct with gRNA-29 targeting *mdoG*	pPF1755 (BsaI-HFv2) + PF5336/PF5337	This study
pPF2703	dCas9(HF1), KmR, OriT, RK2, AraC, PBAD	dCas9 construct with gRNA-31 targeting *rfbB*	pPF1755 (BsaI-HFv2) + PF5340/PF5341	This study
pPF2704	dCas9(HF1), KmR, OriT, RK2, AraC, PBAD	dCas9 construct with gRNA-32 targeting *rfbB*	pPF1755 (BsaI-HFv2) + PF5342/PF5343	This study
pPF2705	dCas9(HF1), KmR, OriT, RK2, AraC, PBAD	dCas9 construct with gRNA-35 targeting CWC46_RS08340	pPF1755 (BsaI-HFv2) + PF5344/PF5345	This study
pPF2706	dCas9(HF1), KmR, OriT, RK2, AraC, PBAD	dCas9 construct with gRNA-36 targeting CWC46_RS08340	pPF1755 (BsaI-HFv2) + PF5346/PF5347	This study
pPF2707	dCas9(HF1), KmR, OriT, RK2, AraC, PBAD	dCas9 construct with gRNA-37 targeting CWC46_RS05635	pPF1755 (BsaI-HFv2) + PF5348/PF5349	This study
pPF2708	dCas9(HF1), KmR, OriT, RK2, AraC, PBAD	dCas9 construct with gRNA-38 targeting CWC46_RS05635	pPF1755 (BsaI-HFv2) + PF5350/PF5351	This study
pPF2709	dCas9(HF1), KmR, OriT, RK2, AraC, PBAD	dCas9 construct with gRNA-41 targeting CWC46_RS04070	pPF1755 (BsaI-HFv2) + PF5352/PF5353	This study
pPF2710	dCas9(HF1), KmR, OriT, RK2, AraC, PBAD	dCas9 construct with gRNA-42 targeting CWC46_RS04070	pPF1755 (BsaI-HFv2) + PF5354/PF5355	This study
pPF2711	dCas9(HF1), KmR, OriT, RK2, AraC, PBAD	dCas9 construct with gRNA-45 targeting *cysC*	pPF1755 (BsaI-HFv2) + PF5356/PF5357	This study
pPF2712	dCas9(HF1), KmR, OriT, RK2, AraC, PBAD	dCas9 construct with gRNA-46 targeting *cysC*	pPF1755 (BsaI-HFv2) + PF5358/PF5359	This study
pPF2713	dCas9(HF1), KmR, OriT, RK2, AraC, PBAD	dCas9 construct with gRNA-47 targeting *rssB*	pPF1755 (BsaI-HFv2) + PF5360/PF5361	This study
pPF2714	dCas9(HF1), KmR, OriT, RK2, AraC, PBAD	dCas9 construct with gRNA-48 targeting *rssB*	pPF1755 (BsaI-HFv2) + PF5362/PF5363	This study
pPF2715	dCas9(HF1), KmR, OriT, RK2, AraC, PBAD	dCas9 construct with gRNA-49 targeting CWC46_RS00475* (labelled as *kdsD* in manuscript if guide was used)	pPF1755 (BsaI-HFv2) + PF5364/PF5365	This study
pPF2716	dCas9(HF1), KmR, OriT, RK2, AraC, PBAD	dCas9 construct with gRNA-50 targeting CWC46_RS00475* (labelled as *kdsD* in manuscript if guide was used)	pPF1755 (BsaI-HFv2) + PF5366/PF5367	This study

Name	Features	Description	Construction	Reference
pPF2717	dCas9(HF1), KmR, OriT, RK2, AraC, PBAD	dCas9 construct with gRNA-51 targeting *kdsD*	pPF1755 (BsaI-HFv2) + PF5368/PF5369	This study
pPF2718	dCas9(HF1), KmR, OriT, RK2, AraC, PBAD	dCas9 construct with gRNA-52 targeting *kdsD*	pPF1755 (BsaI-HFv2) + PF5370/PF5371	This study
pPF2720	dCas9(HF1), KmR, OriT, RK2, AraC, PBAD	dCas9 construct with gRNA-54 targeting *rpoN*	pPF1755 (BsaI-HFv2) + PF5374/PF5375	This study
pPF2721	dCas9(HF1), KmR, OriT, RK2, AraC, PBAD	dCas9 construct with gRNA-55 targeting *trkH*	pPF1755 (BsaI-HFv2) + PF5376/PF5377	This study
pPF2722	dCas9(HF1), KmR, OriT, RK2, AraC, PBAD	dCas9 construct with gRNA-56 targeting *trkH*	pPF1755 (BsaI-HFv2) + PF5378/PF5379	This study
pPF2723	dCas9(HF1), KmR, OriT, RK2, AraC, PBAD	dCas9 construct with gRNA-57 targeting CWC46_RS01810* (labelled as *kdtX* in manuscript if guide was used)	pPF1755 (BsaI-HFv2) + PF5380/PF5381	This study
pPF2724	dCas9(HF1), KmR, OriT, RK2, AraC, PBAD	dCas9 construct with gRNA-58 targeting CWC46_RS01810* (labelled as *kdtX* in manuscript if guide was used)	pPF1755 (BsaI-HFv2) + PF5382/PF5383	This study
pPF2725	dCas9(HF1), KmR, OriT, RK2, AraC, PBAD	dCas9 construct with gRNA-59 targeting *kdtX*	pPF1755 (BsaI-HFv2) + PF5384/PF5385	This study
pPF2726	dCas9(HF1), KmR, OriT, RK2, AraC, PBAD	dCas9 construct with gRNA-60 targeting *kdtX*	pPF1755 (BsaI-HFv2) + PF5386/PF5387	This study
pPF2727	dCas9(HF1), KmR, OriT, RK2, AraC, PBAD	dCas9 construct with gRNA-61 targeting *rfaD* (note: *rfaF* was the only gene significantly enriched in this operon - this guide is labelled as '*rfaF*' in the manuscript)	pPF1755 (BsaI-HFv2) + PF5388/PF5389	This study
pPF2728	dCas9(HF1), KmR, OriT, RK2, AraC, PBAD	dCas9 construct with gRNA-62 targeting *rfaD* (note: *rfaF* was the only gene significantly enriched in this operon - this guide is labelled as '*rfaF*' in the manuscript)	pPF1755 (BsaI-HFv2) + PF5390/PF5391	This study
pPF2729	dCas9(HF1), KmR, OriT, RK2, AraC, PBAD	dCas9 construct with gRNA-65 targeting *ntrBC*	pPF1755 (BsaI-HFv2) + PF5392/PF5393	This study
pPF2730	dCas9(HF1), KmR, OriT, RK2, AraC, PBAD	dCas9 construct with gRNA-66 targeting *ntrBC*	pPF1755 (BsaI-HFv2) + PF5394/PF5395	This study
pPF2731	dCas9(HF1), KmR, OriT, RK2, AraC, PBAD	dCas9 construct with gRNA-67 targeting CWC46_RS03500	pPF1755 (BsaI-HFv2) + PF5396/PF5397	This study
pPF2732	dCas9(HF1), KmR, OriT, RK2, AraC, PBAD	dCas9 construct with gRNA-68 targeting CWC46_RS03500	pPF1755 (BsaI-HFv2) + PF5398/PF5399	This study
pPF2733	dCas9(HF1), KmR, OriT, RK2, AraC, PBAD	dCas9 construct with gRNA-69 targeting *oxyR*	pPF1755 (BsaI-HFv2) + PF5400/PF5401	This study

Name	Features	Description	Construction	Reference
pPF2734	dCas9(HF1), KmR, OriT, RK2, AraC, PBAD	dCas9 construct with gRNA-70 targeting *oxyR*	pPF1755 (BsaI-HFv2) + PF5402/PF5403	This study
pPF2735	dCas9(HF1), KmR, OriT, RK2, AraC, PBAD	dCas9 construct with gRNA-71 targeting *gppA*	pPF1755 (BsaI-HFv2) + PF5404/PF5405	This study
pPF2736	dCas9(HF1), KmR, OriT, RK2, AraC, PBAD	dCas9 construct with gRNA-72 targeting *gppA*	pPF1755 (BsaI-HFv2) + PF5406/PF5407	This study
pPF2737	dCas9(HF1), KmR, OriT, RK2, AraC, PBAD	dCas9 construct with gRNA-73 targeting *thrB*	pPF1755 (BsaI-HFv2) + PF5408/PF5409	This study
pPF2738	dCas9(HF1), KmR, OriT, RK2, AraC, PBAD	dCas9 construct with gRNA-74 targeting *thrB*	pPF1755 (BsaI-HFv2) + PF5410/PF5411	This study
pPF2739	dCas9(HF1), KmR, OriT, RK2, AraC, PBAD	dCas9 construct with gRNA-77 targeting *apaH*	pPF1755 (BsaI-HFv2) + PF5412/PF5413	This study
pPF2740	dCas9(HF1), KmR, OriT, RK2, AraC, PBAD	dCas9 construct with gRNA-78 targeting *apaH*	pPF1755 (BsaI-HFv2) + PF5414/PF5415	This study
pPF2743	dCas9(HF1), KmR, OriT, RK2, AraC, PBAD	dCas9 construct with gRNA-81 targeting *cysJ*	pPF1755 (BsaI-HFv2) + PF5420/PF5421	This study
pPF2744	dCas9(HF1), KmR, OriT, RK2, AraC, PBAD	dCas9 construct with gRNA-82 targeting *cysJ*	pPF1755 (BsaI-HFv2) + PF5422/PF5423	This study
pPF2745	dCas9(HF1), KmR, OriT, RK2, AraC, PBAD	dCas9 construct with gRNA-83 targeting *gmhB*	pPF1755 (BsaI-HFv2) + PF5424/PF5425	This study
pPF2746	dCas9(HF1), KmR, OriT, RK2, AraC, PBAD	dCas9 construct with gRNA-84 targeting *gmhB*	pPF1755 (BsaI-HFv2) + PF5426/PF5427	This study
pPF2747	dCas9(HF1), KmR, OriT, RK2, AraC, PBAD	dCas9 construct with gRNA-85 targeting *apbE*	pPF1755 (BsaI-HFv2) + PF5428/PF5429	This study
pPF2748	dCas9(HF1), KmR, OriT, RK2, AraC, PBAD	dCas9 construct with gRNA-86 targeting *apbE*	pPF1755 (BsaI-HFv2) + PF5430/PF5431	This study
pPF2749	dCas9(HF1), KmR, OriT, RK2, AraC, PBAD	dCas9 construct with gRNA-87 targeting CWC46_RS08390	pPF1755 (BsaI-HFv2) + PF5432/PF5433	This study
pPF2750	dCas9(HF1), KmR, OriT, RK2, AraC, PBAD	dCas9 construct with gRNA-88 targeting CWC46_RS08390	pPF1755 (BsaI-HFv2) + PF5434/PF5435	This study
pPF2757	dCas9(HF1), KmR, OriT, RK2, AraC, PBAD	dCas9 construct with gRNA-95 targeting *nuoI*	pPF1755 (BsaI-HFv2) + PF5448/PF5449	This study
pPF2758	dCas9(HF1), KmR, OriT, RK2, AraC, PBAD	dCas9 construct with gRNA-96 targeting *nuoI*	pPF1755 (BsaI-HFv2) + PF5450/PF5451	This study
pPF2759	dCas9(HF1), KmR, OriT, RK2, AraC, PBAD	dCas9 construct with gRNA-97 targeting *lpxL*	pPF1755 (BsaI-HFv2) + PF5452/PF5453	This study

Name	Features	Description	Construction	Reference
pPF2760	dCas9(HF1), KmR, OriT, RK2, AraC, PBAD	dCas9 construct with gRNA-98 targeting *lpxL*	pPF1755 (BsaI-HFv2) + PF5454/PF5455	This study
pPF2761	dCas9(HF1), KmR, OriT, RK2, AraC, PBAD	dCas9 construct with gRNA-99 targeting CWC46_RS13335	pPF1755 (BsaI-HFv2) + PF5456/PF5457	This study
pPF2762	dCas9(HF1), KmR, OriT, RK2, AraC, PBAD	dCas9 construct with gRNA-100 targeting CWC46_RS13335	pPF1755 (BsaI-HFv2) + PF5458/PF5459	This study
pPF2763	dCas9(HF1), KmR, OriT, RK2, AraC, PBAD	dCas9 construct with gRNA-101 targeting *srfB*	pPF1755 (BsaI-HFv2) + PF5460/PF5461	This study
pPF2764	dCas9(HF1), KmR, OriT, RK2, AraC, PBAD	dCas9 construct with gRNA-102 targeting *srfB*	pPF1755 (BsaI-HFv2) + PF5462/PF5463	This study
pPF2765	dCas9(HF1), KmR, OriT, RK2, AraC, PBAD	dCas9 construct with gRNA-103 targeting *trxB*	pPF1755 (BsaI-HFv2) + PF5464/PF5465	This study
pPF2766	dCas9(HF1), KmR, OriT, RK2, AraC, PBAD	dCas9 construct with gRNA-104 targeting *trxB*	pPF1755 (BsaI-HFv2) + PF5466/PF5467	This study
pPF2769	dCas9(HF1), KmR, OriT, RK2, AraC, PBAD	dCas9 construct with gRNA-107 targeting CWC46_RS17860	pPF1755 (BsaI-HFv2) + PF5472/PF5473	This study
pPF2770	dCas9(HF1), KmR, OriT, RK2, AraC, PBAD	dCas9 construct with gRNA-108 targeting CWC46_RS17860	pPF1755 (BsaI-HFv2) + PF5474/PF5475	This study
pPF2771	dCas9(HF1), KmR, OriT, RK2, AraC, PBAD	dCas9 construct with gRNA-109 targeting *apt*	pPF1755 (BsaI-HFv2) + PF5476/PF5477	This study
pPF2772	dCas9(HF1), KmR, OriT, RK2, AraC, PBAD	dCas9 construct with gRNA-110 targeting *apt*	pPF1755 (BsaI-HFv2) + PF5478/PF5479	This study
pPF2775	dCas9(HF1), KmR, OriT, RK2, AraC, PBAD	dCas9 construct with gRNA-113 targeting *rppH*	pPF1755 (BsaI-HFv2) + PF5484/PF5485	This study
pPF2779	dCas9(HF1), KmR, OriT, RK2, AraC, PBAD	dCas9 construct with gRNA-117 targeting *rnfH*	pPF1755 (BsaI-HFv2) + PF5492/PF5493	This study
pPF2780	dCas9(HF1), KmR, OriT, RK2, AraC, PBAD	dCas9 construct with gRNA-118 targeting *rnfH*	pPF1755 (BsaI-HFv2) + PF5494/PF5495	This study
pPF2781	dCas9(HF1), KmR, OriT, RK2, AraC, PBAD	dCas9 construct with gRNA-119 targeting *rlmE*	pPF1755 (BsaI-HFv2) + PF5496/PF5497	This study
pPF2782	dCas9(HF1), KmR, OriT, RK2, AraC, PBAD	dCas9 construct with gRNA-120 targeting *rlmE*	pPF1755 (BsaI-HFv2) + PF5498/PF5499	This study
pPF2783	dCas9(HF1), KmR, OriT, RK2, AraC, PBAD	dCas9 construct with gRNA-121 targeting *clpP*	pPF1755 (BsaI-HFv2) + PF5500/PF5501	This study
pPF2784	dCas9(HF1), KmR, OriT, RK2, AraC, PBAD	dCas9 construct with gRNA-122 targeting *clpP*	pPF1755 (BsaI-HFv2) + PF5502/PF5503	This study

Name	Features	Description	Construction	Reference
pPF2785	dCas9(HF1), KmR, OriT, RK2, AraC, PBAD	dCas9 construct with gRNA-123 targeting *ompW*	pPF1755 (BsaI-HFv2) + PF5504/PF5505	This study
pPF2786	dCas9(HF1), KmR, OriT, RK2, AraC, PBAD	dCas9 construct with gRNA-124 targeting scrambled guide	pPF1755 (BsaI-HFv2) + PF5506/PF5507	This study

*Guide targeting start of operon, but gene not enriched in Tn-seq screen

Table S3. Oligonucleotides used in this study.

Name	Sequence	Description
PF5300	**TGCA**GTTCAATAAATACTCTCCAT	dCas9 gRNA-5 targeting *flhD*_BsaI overhangs fwd
PF5267	**AAAC**ATGGAGAGTATTTATTGAAC	dCas9 gRNA-5 targeting *flhD*_BsaI overhangs rev
PF5268	**TGCA**AAGTACCCATAATCTCATCC	dCas9 gRNA-6 targeting *flhD*_BsaI overhangs fwd
PF5269	**AAAC**GGATGAGATTATGGGTACTT	dCas9 gRNA-6 targeting *flhD*_BsaI overhangs rev
PF5270	**TGCA**ATTAATACCAAGGCGAAACA	dCas9 gRNA-7 targeting *flhD*_BsaI overhangs fwd
PF5271	**AAAC**TGTTTCGCCTTGGTATTAAT	dCas9 gRNA-7 targeting *flhD*_BsaI overhangs rev
PF5272	**TGCA**AATTAACGATGAAAAAGCAT	dCas9 gRNA-8 targeting *flhD*_BsaI overhangs fwd
PF5273	**AAAC**ATGCTTTTTCATCGTTAATT	dCas9 gRNA-8 targeting *flhD*_BsaI overhangs rev
PF5274	**TGCA**TTATCTCCATCTAACAGCCC	dCas9 gRNA-9 targeting *igaA*_BsaI overhangs fwd
PF5275	**AAAC**GGGCTGTTAGATGGAGATAA	dCas9 gRNA-9 targeting *igaA*_BsaI overhangs rev
PF5316	**TGCA**CAGTGCCTAAGAAATAAAAC	dCas9 gRNA-11 targeting *rsxA*_BsaI overhangs fwd
PF5317	**AAAC**GTTTTATTTCTTAGGCACTG	dCas9 gRNA-11 targeting *rsxA*_BsaI overhangs rev
PF5318	**TGCA**AACGAAGTTATTGACCAGGA	dCas9 gRNA-12 targeting *rsxA*_BsaI overhangs fwd
PF5319	**AAAC**TCCTGGTCAATAACTTCGTT	dCas9 gRNA-12 targeting *rsxA*_BsaI overhangs rev
PF5320	**TGCA**AGTTATTTTTCCGGACATAC	dCas9 gRNA-15 targeting *sapA*_BsaI overhangs fwd
PF5321	**AAAC**GTATGTCCGGAAAAATAACT	dCas9 gRNA-15 targeting *sapA*_BsaI overhangs rev
PF5322	**TGCA**TGGTACCGGTACGGCCAGCG	dCas9 gRNA-16 targeting *sapA*_BsaI overhangs fwd
PF5323	**AAAC**CGCTGGCCGTACCGGTACCA	dCas9 gRNA-16 targeting *sapA*_BsaI overhangs rev
PF5324	**TGCA**GCTCTCGGGTGATTAGCCAT	dCas9 gRNA-21 targeting CWC46_RS18195_BsaI overhangs fwd
PF5325	**AAAC**ATGGCTAATCACCCGAGAGC	dCas9 gRNA-21 targeting CWC46_RS18195_BsaI overhangs rev
PF5326	**TGCA**GCTTTGCCGGGTGTGTTGTC	dCas9 gRNA-22 targeting CWC46_RS18195_BsaI overhangs fwd
PF5327	**AAAC**GACAACACACCCGGCAAAGC	dCas9 gRNA-22 targeting CWC46_RS18195_BsaI overhangs rev
PF5328	**TGCA**TCAAGAGTCGGGTCTGTTTG	dCas9 gRNA-23 targeting *crp*_BsaI overhangs fwd
PF5329	**AAAC**CAAACAGACCCGACTCTTGA	dCas9 gRNA-23 targeting *crp*_BsaI overhangs rev

Name	Sequence	Description
PF5330	**TGCA**TGTGAATATGGCAATGAGAA	dCas9 gRNA-24 targeting *crp*_BsaI overhangs fwd
PF5331	**AAAC**TTCTCATTGCCATATTCACA	dCas9 gRNA-24 targeting *crp*_BsaI overhangs rev
PF5332	**TGCA**AATAATAAGCTTTATTTATC	dCas9 gRNA-25 targeting *galU*_BsaI overhangs fwd
PF5333	**AAAC**GATAAATAAAGCTTATTATT	dCas9 gRNA-25 targeting *galU*_BsaI overhangs rev
PF5334	**TGCA**TCGTGTTCCAAGTCCAGCCA	dCas9 gRNA-26 targeting *galU*_BsaI overhangs fwd
PF5335	**AAAC**TGGCTGGACTTGGAACACGA	dCas9 gRNA-26 targeting *galU*_BsaI overhangs rev
PF5336	**TGCA**CCACTGCTGAAAGCCAGCGA	dCas9 gRNA-29 targeting *mdoG*_BsaI overhangs fwd
PF5337	**AAAC**TCGCTGGCTTTCAGCAGTGG	dCas9 gRNA-29 targeting *mdoG*_BsaI overhangs rev
PF5340	**TGCA**CATCCAATGACGAAATTGCT	dCas9 gRNA-31 targeting *rfbB*_BsaI overhangs fwd
PF5341	**AAAC**AGCAATTTCGTCATTGGATG	dCas9 gRNA-31 targeting *rfbB*_BsaI overhangs rev
PF5342	**TGCA**TACCGAGGAACCAATAAAAC	dCas9 gRNA-32 targeting *rfbB*_BsaI overhangs fwd
PF5343	**AAAC**GTTTTATTGGTTCCTCGGTA	dCas9 gRNA-32 targeting *rfbB*_BsaI overhangs rev
PF5344	**TGCA**CCCGCTATTGATTTTAATGA	dCas9 gRNA-35 targeting CWC46_RS08340 _BsaI overhangs fwd
PF5345	**AAAC**TCATTAAAATCAATAGCGGG	dCas9 gRNA-35 targeting CWC46_RS08340_BsaI overhangs rev
PF5346	**TGCA**GAAAGCGCCTGTGATACGTC	dCas9 gRNA-36 targeting CWC46_RS08340 _BsaI overhangs fwd
PF5347	**AAAC**GACGTATCACAGGCGCTTTC	dCas9 gRNA-36 targeting CWC46_RS08340_BsaI overhangs rev
PF5348	**TGCA**GGTGTCTTTCTTGATATTGA	dCas9 gRNA-37 targeting CWC46_RS05635_BsaI overhangs fwd
PF5349	**AAAC**TCAATATCAAGAAAGACACC	dCas9 gRNA-37 targeting CWC46_RS05635_BsaI overhangs rev
PF5350	**TGCA**TTCCAGCAGCATGGCAAAGC	dCas9 gRNA-38 targeting CWC46_RS05635_BsaI overhangs fwd
PF5351	**AAAC**GCTTTGCCATGCTGCTGGAA	dCas9 gRNA-38 targeting CWC46_RS05635_BsaI overhangs rev
PF5352	**TGCA**GAGCACGATCAACACGTAGT	dCas9 gRNA-41 targeting CWC46_RS04070_BsaI overhangs fwd
PF5353	**AAAC**ACTACGTGTTGATCGTGCTC	dCas9 gRNA-41 targeting CWC46_RS04070_BsaI overhangs rev
PF5354	**TGCA**ACTGTAAACCTGCTGAAAAA	dCas9 gRNA-42 targeting CWC46_RS04070_BsaI overhangs fwd
PF5355	**AAAC**TTTTTCAGCAGGTTTACAGT	dCas9 gRNA-42 targeting CWC46_RS04070_BsaI overhangs rev
PF5356	**TGCA**CGTCAGCAGACGCCATCACT	dCas9 gRNA-45 targeting *cysC*_BsaI overhangs fwd
PF5357	**AAAC**AGTGATGGCGTCTGCTGACG	dCas9 gRNA-45 targeting *cysC*_BsaI overhangs rev

Name	Sequence	Description
PF5358	**TGCA**TCAGTTCGTGTAACCGCATG	dCas9 gRNA-46 targeting *cysC*_BsaI overhangs fwd
PF5359	**AAAC**CATGCGGTTACACGAACTGA	dCas9 gRNA-46 targeting *cysC*_BsaI overhangs rev
PF5360	**TGCA**CCCGTTAGTGGTTGTGCCAT	dCas9 gRNA-47 targeting *rssB*_BsaI overhangs fwd
PF5361	**AAAC**ATGGCACAACCACTAACGGG	dCas9 gRNA-47 targeting *rssB*_BsaI overhangs rev
PF5362	**TGCA**AATAACCCGCCAGCACAGAA	dCas9 gRNA-48 targeting *rssB*_BsaI overhangs fwd
PF5363	**AAAC**TTCTGTGCTGGCGGGTTATT	dCas9 gRNA-48 targeting *rssB*_BsaI overhangs rev
PF5364	**TGCA**CTTTATTCAGGTATAATCAC	dCas9 gRNA-49 targeting CWC46_RS00475_BsaI overhangs fwd
PF5365	**AAAC**GTGATTATACCTGAATAAAG	dCas9 gRNA-49 targeting CWC46_RS00475_BsaI overhangs rev
PF5366	**TGCA**GCATGCAGCGTCCTTTATTC	dCas9 gRNA-50 targeting CWC46_RS00475_BsaI overhangs fwd
PF5367	**AAAC**GAATAAAGGACGCTGCATGC	dCas9 gRNA-50 targeting CWC46_RS00475_BsaI overhangs rev
PF5368	**TGCA**GTAGCTCATAATGTAACATG	dCas9 gRNA-51 targeting *kdsD*_BsaI overhangs fwd
PF5369	**AAAC**CATGTTACATTATGAGCTAC	dCas9 gRNA-51 targeting *kdsD*_BsaI overhangs rev
PF5370	**TGCA**GCTTGTTGGAAATCGAAACC	dCas9 gRNA-52 targeting *kdsD*_BsaI overhangs fwd
PF5371	**AAAC**GGTTTCGATTTCCAACAAGC	dCas9 gRNA-52 targeting *kdsD*_BsaI overhangs rev
PF5374	**TGCA**CTGTTGCAGTTGTGGTGTCA	dCas9 gRNA-54 targeting *rpoN*_BsaI overhangs fwd
PF5375	**AAAC**TGACACCACAACTGCAACAG	dCas9 gRNA-54 targeting *rpoN*_BsaI overhangs rev
PF5376	**TGCA**CATTACAGATGCCTTCCTTA	dCas9 gRNA-55 targeting *trkH*_BsaI overhangs fwd
PF5377	**AAAC**TAAGGAAGGCATCTGTAATG	dCas9 gRNA-55 targeting *trkH*_BsaI overhangs rev
PF5378	**TGCA**CAGGCCAACAATGCGGGTTA	dCas9 gRNA-56 targeting *trkH*_BsaI overhangs fwd
PF5379	**AAAC**TAACCCGCATTGTTGGCCTG	dCas9 gRNA-56 targeting *trkH*_BsaI overhangs rev
PF5380	**TGCA**CGAATTCTATATATTTACAG	dCas9 gRNA-57 targeting CWC46_RS01810_BsaI overhangs fwd
PF5381	**AAAC**CTGTAAATATATAGAATTCG	dCas9 gRNA-57 targeting CWC46_RS01810_BsaI overhangs rev
PF5382	**TGCA**CCCAGATCAACGGCTGGATA	dCas9 gRNA-58 targeting CWC46_RS01810_BsaI overhangs fwd
PF5383	**AAAC**TATCCAGCCGTTGATCTGGG	dCas9 gRNA-58 targeting CWC46_RS01810_BsaI overhangs rev
PF5384	**TGCA**CCAACAAGTCTGCGGCATTG	dCas9 gRNA-59 targeting *kdtX*_BsaI overhangs fwd
PF5385	**AAAC**CAATGCCGCAGACTTGTTGG	dCas9 gRNA-59 targeting *kdtX*_BsaI overhangs rev

Name	Sequence	Description
PF5386	**TGCA**CAGCCCATGCTACAGAAGAA	dCas9 gRNA-60 targeting *kdtX*_BsaI overhangs fwd
PF5387	**AAAC**TTCTTCTGTAGCATGGGCTG	dCas9 gRNA-60 targeting *kdtX*_BsaI overhangs rev
PF5388	**TGCA**ATTACCCTTGTTAAACTTGC	dCas9 gRNA-61 targeting *rfaF*_BsaI overhangs fwd
PF5389	**AAAC**GCAAGTTTAACAAGGGTAAT	dCas9 gRNA-61 targeting *rfaF*_BsaI overhangs rev
PF5390	**TGCA**ACCGCCAGTAACGATAATCA	dCas9 gRNA-62 targeting *rfaF*_BsaI overhangs fwd
PF5391	**AAAC**TGATTATCGTTACTGGCGGT	dCas9 gRNA-62 targeting *rfaF*_BsaI overhangs rev
PF5392	**TGCA**ATTGAGGATCTGCCCAGCAT	dCas9 gRNA-65 targeting *ntrBC*_BsaI overhangs fwd
PF5393	**AAAC**ATGCTGGGCAGATCCTCAAT	dCas9 gRNA-65 targeting *ntrBC*_BsaI overhangs rev
PF5394	**TGCA**TACTGTTAATCAAAGAATTG	dCas9 gRNA-66 targeting *ntrBC*_BsaI overhangs fwd
PF5395	**AAAC**CAATTCTTTGATTAACAGTA	dCas9 gRNA-66 targeting *ntrBC*_BsaI overhangs rev
PF5396	**TGCA**GATGTCGCATTTTTTCCTCC	dCas9 gRNA-67 targeting CWC46_RS03500_BsaI overhangs fwd
PF5397	**AAAC**GGAGGAAAAAATGCGACATC	dCas9 gRNA-67 targeting CWC46_RS03500_BsaI overhangs rev
PF5398	**TGCA**CTTCCAGTTGCCCATAACTA	dCas9 gRNA-68 targeting CWC46_RS03500_BsaI overhangs fwd
PF5399	**AAAC**TAGTTATGGGCAACTGGAAG	dCas9 gRNA-68 targeting CWC46_RS03500_BsaI overhangs rev
PF5400	**TGCA**TCGACGAAAATGACGATGCT	dCas9 gRNA-69 targeting *oxyR*_BsaI overhangs fwd
PF5401	**AAAC**AGCATCGTCATTTTCGTCGA	dCas9 gRNA-69 targeting *oxyR*_BsaI overhangs rev
PF5402	**TGCA**TGTGGGTTGGCTAACGTGAC	dCas9 gRNA-70 targeting *oxyR*_BsaI overhangs fwd
PF5403	**AAAC**GTCACGTTAGCCAACCCACA	dCas9 gRNA-70 targeting *oxyR*_BsaI overhangs rev
PF5404	**TGCA**AGCGAAGAAGAACTTGGCAT	dCas9 gRNA-71 targeting *gppA*_BsaI overhangs fwd
PF5405	**AAAC**ATGCCAAGTTCTTCTTCGCT	dCas9 gRNA-71 targeting *gppA*_BsaI overhangs rev
PF5406	**TGCA**CACCAACATGTGAAAGCTGT	dCas9 gRNA-72 targeting *gppA*_BsaI overhangs fwd
PF5407	**AAAC**ACAGCTTTCACATGTTGGTG	dCas9 gRNA-72 targeting *gppA*_BsaI overhangs rev
PF5408	**TGCA**AAAGCCCGCACTGTAAGGTG	dCas9 gRNA-73 targeting *thrB*_BsaI overhangs fwd
PF5409	**AAAC**CACCTTACAGTGCGGGCTTT	dCas9 gRNA-73 targeting *thrB*_BsaI overhangs rev
PF5410	**TGCA**TGCATTGCTTTCAATAATGT	dCas9 gRNA-74 targeting *thrB*_BsaI overhangs fwd
PF5411	**AAAC**ACATTATTGAAAGCAATGCA	dCas9 gRNA-74 targeting *thrB*_BsaI overhangs rev

Name	Sequence	Description
PF5412	**TGCA**AGCCACAAGGTGTCCTGTGT	dCas9 gRNA-77 targeting *apaH*_BsaI overhangs fwd
PF5413	**AAAC**ACACAGGACACCTTGTGGCT	dCas9 gRNA-77 targeting *apaH*_BsaI overhangs rev
PF5414	**TGCA**CAGATCGCCAGTCAGCCACA	dCas9 gRNA-78 targeting *apaH*_BsaI overhangs fwd
PF5415	**AAAC**TGTGGCTGACTGGCGATCTG	dCas9 gRNA-78 targeting *apaH*_BsaI overhangs rev
PF5420	**TGCA**TGATATCTGTATCCTTGTTC	dCas9 gRNA-81 targeting *cysJ*_BsaI overhangs fwd
PF5421	**AAAC**GAACAAGGATACAGATATCA	dCas9 gRNA-81 targeting *cysJ*_BsaI overhangs rev
PF5422	**TGCA**CTCAACGGGAGCAACGAAGT	dCas9 gRNA-82 targeting *cysJ*_BsaI overhangs fwd
PF5423	**AAAC**ACTTCGTTGCTCCCGTTGAG	dCas9 gRNA-82 targeting *cysJ*_BsaI overhangs rev
PF5424	**TGCA**TCACGGTCAAGAAAGATTGC	dCas9 gRNA-83 targeting *gmhB*_BsaI overhangs fwd
PF5425	**AAAC**GCAATCTTTCTTGACCGTGA	dCas9 gRNA-83 targeting *gmhB*_BsaI overhangs rev
PF5426	**TGCA**CAACATTTATGGTACCGTCA	dCas9 gRNA-84 targeting *gmhB*_BsaI overhangs fwd
PF5427	**AAAC**TGACGGTACCATAAATGTTG	dCas9 gRNA-84 targeting *gmhB*_BsaI overhangs rev
PF5428	**TGCA**CCAGCAGATTTGCTTCGACA	dCas9 gRNA-85 targeting *apbE*_BsaI overhangs fwd
PF5429	**AAAC**TGTCGAAGCAAATCTGCTGG	dCas9 gRNA-85 targeting *apbE*_BsaI overhangs rev
PF5430	**TGCA**CCCGGTTAATATCCAGACAA	dCas9 gRNA-86 targeting *apbE*_BsaI overhangs fwd
PF5431	**AAAC**TTGTCTGGATATTAACCGGG	dCas9 gRNA-86 targeting *apbE*_BsaI overhangs rev
PF5432	**TGCA**CCTGTTAATTCCTGTCTATG	dCas9 gRNA-87 targeting CWC46_08390_BsaI overhangs fwd
PF5433	**AAAC**CATAGACAGGAATTAACAGG	dCas9 gRNA-87 targeting CWC46_08390_BsaI overhangs rev
PF5434	**TGCA**AATTTGGCTTTCAATTCATC	dCas9 gRNA-88 targeting CWC46_08390_BsaI overhangs fwd
PF5435	**AAAC**GATGAATTGAAAGCCAAATT	dCas9 gRNA-88 targeting CWC46_08390_BsaI overhangs rev
PF5448	**TGCA**CACTAGCTCTTTCAATGTCA	dCas9 gRNA-95 targeting *nuoI*_BsaI overhangs fwd
PF5449	**AAAC**TGACATTGAAAGAGCTAGTG	dCas9 gRNA-95 targeting *nuoI*_BsaI overhangs rev
PF5450	**TGCA**CCATGCAGATACTGCGAATC	dCas9 gRNA-96 targeting *nuoI*_BsaI overhangs fwd
PF5451	**AAAC**GATTCGCAGTATCTGCATGG	dCas9 gRNA-96 targeting *nuoI*_BsaI overhangs rev
PF5452	**TGCA**ATGGTCTCAAGTATCAAAAT	dCas9 gRNA-97 targeting *lpxL*_BsaI overhangs fwd
PF5453	**AAAC**ATTTTGATACTTGAGACCAT	dCas9 gRNA-97 targeting *lpxL*_BsaI overhangs rev

Name	Sequence	Description
PF5454	**TGCA**AAATGAAGGGATTTGTGTCA	dCas9 gRNA-98 targeting *lpxL*_BsaI overhangs fwd
PF5455	**AAAC**TGACACAAATCCCTTCATTT	dCas9 gRNA-98 targeting *lpxL*_BsaI overhangs rev
PF5456	**TGCA**CATAGGTATTCCATCAGCAC	dCas9 gRNA-99 targeting CWC46_RS13335_BsaI overhangs fwd
PF5457	**AAAC**GTGCTGATGGAATACCTATG	dCas9 gRNA-99 targeting CWC46_RS13335_BsaI overhangs rev
PF5458	**TGCA**AAATCAGCAATAGAACTCAT	dCas9 gRNA-100 targeting CWC46_RS13335_BsaI overhangs fwd
PF5459	**AAAC**ATGAGTTCTATTGCTGATTT	dCas9 gRNA-100 targeting CWC46_RS13335_BsaI overhangs rev
PF5460	**TGCA**CCTTTTGTTATAAACAGCCA	dCas9 gRNA-101 targeting *srfB*_BsaI overhangs fwd
PF5461	**AAAC**TGGCTGTTTATAACAAAAGG	dCas9 gRNA-101 targeting *srfB*_BsaI overhangs rev
PF5462	**TGCA**AAATATTTTTGTCACGCCAA	dCas9 gRNA-102 targeting *srfB*_BsaI overhangs fwd
PF5463	**AAAC**TTGGCGTGACAAAAATATTT	dCas9 gRNA-102 targeting *srfB*_BsaI overhangs rev
PF5464	**TGCA**AGTTCCTCAATTCATTTGAA	dCas9 gRNA-103 targeting *trxB*_BsaI overhangs fwd
PF5465	**AAAC**TTCAAATGAATTGAGGAACT	dCas9 gRNA-103 targeting *trxB*_BsaI overhangs rev
PF5466	**TGCA**TAATAATTTACGGTGTTTTA	dCas9 gRNA-104 targeting *trxB*_BsaI overhangs fwd
PF5467	**AAAC**TAAAACACCGTAAATTATTA	dCas9 gRNA-104 targeting *trxB*_BsaI overhangs rev
PF5472	**TGCA**ACCGATAAACCCGGCCGCAC	dCas9 gRNA-107 targeting CWC46_RS17860_BsaI overhangs fwd
PF5473	**AAAC**GTGCGGCCGGGTTTATCGGT	dCas9 gRNA-107 targeting CWC46_RS17860_BsaI overhangs rev
PF5474	**TGCA**TACATAAAAACCGATAAACC	dCas9 gRNA-108 targeting CWC46_RS17860_BsaI overhangs fwd
PF5475	**AAAC**GGTTTATCGGTTTTTATGTA	dCas9 gRNA-108 targeting CWC46_RS17860_BsaI overhangs rev
PF5476	**TGCA**TAATTCTGGTTGCAGTGATA	dCas9 gRNA-109 targeting *apt*_BsaI overhangs fwd
PF5477	**AAAC**TATCACTGCAACCAGAATTA	dCas9 gRNA-109 targeting *apt*_BsaI overhangs rev
PF5478	**TGCA**ATACTATTTTTAATTAATTC	dCas9 gRNA-110 targeting *apt*_BsaI overhangs fwd
PF5479	**AAAC**GAATTAATTAAAAATAGTAT	dCas9 gRNA-110 targeting *apt*_BsaI overhangs rev
PF5484	**TGCA**TAGCCATCATCATCGATCAC	dCas9 gRNA-113 targeting *rppH*_BsaI overhangs fwd
PF5485	**AAAC**GTGATCGATGATGATGGCTA	dCas9 gRNA-113 targeting *rppH*_BsaI overhangs rev
PF5492	**TGCA**CAATGCCGATCGATTTATCT	dCas9 gRNA-117 targeting *rnfH*_BsaI overhangs fwd
PF5493	**AAAC**AGATAAATCGATCGGCATTG	dCas9 gRNA-117 targeting *rnfH*_BsaI overhangs rev

Name	Sequence	Description
PF5494	**TGCA**TACATCTGCTCCGCACTGAA	dCas9 gRNA-118 targeting *rnfH*_BsaI overhangs fwd
PF5495	**AAAC**TTCAGTGCGGAGCAGATGTA	dCas9 gRNA-118 targeting *rnfH*_BsaI overhangs rev
PF5496	**TGCA**GTGTTCCTGCAACCAGCGGC	dCas9 gRNA-119 targeting *rlmE*_BsaI overhangs fwd
PF5497	**AAAC**GCCGCTGGTTGCAGGAACAC	dCas9 gRNA-119 targeting *rlmE*_BsaI overhangs rev
PF5498	**TGCA**TAAAGTGTTCCTGCAACCAG	dCas9 gRNA-120 targeting *rlmE*_BsaI overhangs fwd
PF5499	**AAAC**CTGGTTGCAGGAACACTTTA	dCas9 gRNA-120 targeting *rlmE*_BsaI overhangs rev
PF5500	**TGCA**GTCTCCTAGATAAATACATT	dCas9 gRNA-121 targeting *clpP*_BsaI overhangs fwd
PF5501	**AAAC**AATGTATTTATCTAGGAGAC	dCas9 gRNA-121 targeting *clpP*_BsaI overhangs rev
PF5502	**TGCA**ATCGGCACCAGAGCCATATG	dCas9 gRNA-122 targeting *clpP*_BsaI overhangs fwd
PF5503	**AAAC**CATATGGCTCTGGTGCCGAT	dCas9 gRNA-122 targeting *clpP*_BsaI overhangs rev
PF5504	**TGCA**CATTACCCACCCCTTTTGTA	dCas9 gRNA-123 targeting *ompW*_BsaI overhangs fwd
PF5505	**AAAC**TACAAAAGGGGTGGGTAATG	dCas9 gRNA-123 targeting *ompW*_BsaI overhangs rev
PF5506	**TGCA**GCTGCTCAAGCGTCACCCCG	dCas9 gRNA-124 targeting scrambled guide_BsaI overhangs fwd
PF5507	**AAAC**CGGGGTGACGCTTGAGCAGC	dCas9 gRNA-124 targeting scrambled guide_BsaI overhangs rev
PF5508	CCACTCTTATCCATCAATCCATC	gRNA screening in dCas9 construct fwd
PF5509	GAGTCCAAGACTAGTAAGCTTC	gRNA screening in dCas9 construct rev

Table S4. Transposon sequencing insertion table.

File	Total Reads	Reads Matched	% Matched	Reads Mapped	% Mapped	Unique Insertion Sites: NZ_CP025085.1	Seq Len/UIS: NZ_CP025085.1	Total Unique Insertion Sites	Total Seq Len/Total UIS
6439 all files combined	10921528	10253596	93.9	9123414	89.0	353093	14.1	353093	14.1
6439 control combined	8979128	8467507	94.3	7446016	87.9	283677	17.5	283677	17.5
6439 MOI1 combined	1942400	1786089	92.0	1561415	87.4	130619	38.1	130619	38.1
Control 1 (6439-01-00-01_S1_L001_R1_001.trimmed.fastq)	1697045	1601792	94.4	1475816	92.1	77516	64.1	77516	64.1
Control 2 (6439-02-00-01_S2_L001_R1_001.trimmed.fastq)	5058996	4791838	94.7	4280640	89.3	139354	35.7	139354	35.7
Control 3 (6439-03-00-01_S3_L001_R1_001.trimmed.fastq)	2223087	2073877	93.3	1791717	86.4	183774	27.1	183774	27.1
MOI 1 (6439-04-00-01_S4_L001_R1_001.trimmed.fastq)	554969	518484	93.4	474937	91.6	33990	146.3	33990	146.3
MOI 2 (6439-05-00-01_S5_L001_R1_001.trimmed.fastq)	791480	727712	91.9	631619	86.8	79260	62.7	79260	62.7
MOI 3 6439-06-00-01_S6_L001_R1_001.trimmed.fastq)	595951	539893	90.6	468687	86.8	64446	77.1	64446	77.1

Table S5. Serratia sp. *Serratia sp.* ATCC 39006 genes significantly enriched in transposon sequencing after jumbo phage PCH45 challenge.

Locus tag	Gene	Product	logCPM	log2FC MOI1	PValue MOI1	padj MOI1
CWC46_RS11475	*flgK*	flagellar hook-associated protein FlgK	13.771871	5.281709	6.93E-65	5.09E-61
CWC46_RS11480	*flgL*	flagellar hook-filament junction protein FlgL	13.516914	5.481931	1.29E-63	3.93E-60
CWC46_RS11550	*fliE*	flagellar hook-basal body complex protein FliE	12.627544	5.321740	1.60E-63	3.93E-60
CWC46_RS11485	*fliR*	flagellar type III secretion system protein FliR	15.816385	5.198278	2.01E-62	3.70E-59
CWC46_RS11490	*fliQ*	flagellar biosynthetic protein FliQ	12.411390	5.331365	2.62E-62	3.85E-59
CWC46_RS11350	*flhC*	flagellar transcriptional regulator FlhC	14.860187	5.323722	1.23E-60	1.50E-57
CWC46_RS11345	*flhD*	flagellar transcriptional regulator FlhD	13.665137	5.326991	4.89E-60	5.14E-57
CWC46_RS11495	*fliP*	flagellar biosynthetic protein FliP	12.184277	5.123742	1.78E-58	1.64E-55
CWC46_RS11575		hypothetical protein	14.629586	4.984860	2.14E-58	1.75E-55
CWC46_RS11530	*fliI*	flagellum-specific ATP synthase FliL	11.827702	5.155880	3.78E-57	2.78E-54
CWC46_RS11440	*flgD*	flagellar hook assembly protein FlgD	12.116721	5.013551	1.94E-56	1.30E-53
CWC46_RS11400	*flhB*	flagellar type III secretion system protein FlhB	12.105850	5.118192	1.34E-55	7.59E-53
CWC46_RS11445	*flgE*	flagellar hook protein FlgE	12.195326	5.022657	1.34E-55	7.59E-53
CWC46_RS11610	*fliA*	RNA polymerase sigma factor FliA	13.424568	5.142763	1.72E-55	9.05E-53
CWC46_RS11425	*flgA*	flagellar basal body P-ring formation protein FlgA	11.162383	5.353385	2.03E-55	9.93E-53
CWC46_RS21680	*rfbA*	glucose-1-phosphate thymidylyltransferase	10.657462	4.767958	2.88E-54	1.24E-51
CWC46_RS11510	*fliM*	flagellar motor switch protein FliM	11.506650	5.133322	9.95E-54	4.06E-51
CWC46_RS11540	*fliG*	flagellar motor switch protein FliG	11.253025	5.002264	2.77E-53	1.07E-50
CWC46_RS11570	*fliC*	flagellin FliC	11.504294	4.977913	6.56E-53	2.41E-50
CWC46_RS11525	*fliJ*	flagella biosynthesis chaperone FliJ	10.283525	5.170055	2.28E-52	7.99E-50
CWC46_RS11545	*fliF*	flagellar basal body M-ring protein FliF	12.125111	4.936041	7.34E-52	2.45E-49
CWC46_RS11565	*fliD*	flagellar filament capping protein FliD	10.680203	5.173475	2.52E-51	8.06E-49
CWC46_RS11405	*flhA*	flagellar biosynthesis protein FlhA	11.911225	5.199379	4.06E-51	1.24E-48
CWC46_RS21670	*rfbC*	dTDP-4-dehydrorhamnose 3,5-epimerase	11.959144	4.561260	3.57E-50	1.05E-47
CWC46_RS11460	*flgH*	flagellar basal body L-ring protein FlgH	10.962719	5.656860	1.20E-49	3.27E-47

Locus tag	Gene	Product	logCPM	log2FC MOI1	PValue MOI1	padj MOI1
CWC46_RS11360	*motB*	motility protein MotB	10.759462	4.751031	1.12E-47	2.94E-45
CWC46_RS21675	*rfbD*	dTDP-4-dehydrorhamnose reductase	11.096805	5.069712	1.94E-47	4.92E-45
CWC46_RS11435	*flgC*	flagellar basal body rod protein FlgC	10.714543	5.495830	2.28E-47	5.58E-45
CWC46_RS11415	*flgN*	FlgN family protein	10.203332	5.310393	3.43E-47	8.14E-45
CWC46_RS11505	*fliN*	flagellar motor switch protein FliN	10.555705	4.759302	1.04E-46	2.39E-44
CWC46_RS11465	*flgI*	flagellar basal body P-ring protein FlgI	11.576204	5.394851	4.45E-46	9.62E-44
CWC46_RS11355	*motA*	flagellar motor stator protein MotA	11.639618	5.239931	9.13E-45	1.92E-42
CWC46_RS21685	*rfbB*	dTDP-glucose 4,6-dehydratase	10.454887	4.426906	1.20E-44	2.44E-42
CWC46_RS11520	*fliK*	flagellar hook-length control protein FliK	11.998348	4.736919	1.09E-40	2.17E-38
CWC46_RS11535	*fliH*	flagellar assembly protein FliH	10.473689	5.356459	1.32E-40	2.48E-38
CWC46_RS11705	*mdoH*	glucans biosynthesis glucosyltransferase MdoH	11.162143	4.785993	1.32E-40	2.48E-38
CWC46_RS13965	*rssB*	two-component system response regulator RssB	11.677837	4.012901	1.36E-39	2.50E-37
CWC46_RS11500	*fliO*	flagellar biosynthetic protein FliO	10.887862	5.066250	2.54E-39	4.55E-37
CWC46_RS11515	*fliL*	flagellar basal body-associated protein FliL	10.130956	5.063314	4.54E-38	7.95E-36
CWC46_RS11700	*mdoG*	glucan biosynthesis protein G	10.952551	4.702907	1.18E-37	2.01E-35
CWC46_RS11455	*flgG*	flagellar basal-body rod protein FlgG	11.185338	5.890731	3.77E-36	6.29E-34
CWC46_RS11450	*flgF*	flagellar basal body rod protein FlgF	11.239393	5.319463	6.98E-30	1.09E-27
CWC46_RS00620	*igaA*	intracellular growth attenuator family protein	7.865269	7.239694	1.51E-28	2.27E-26
CWC46_RS11470	*flgJ*	flagellar assembly peptidoglycan hydrolase FlgJ	10.008146	3.415003	3.41E-27	5.01E-25
CWC46_RS11430	*flgB*	flagellar basal body rod protein FlgB	10.851853	5.256637	9.29E-23	1.29E-20
CWC46_RS13960	*galU*	UTP-glucose-1-phosphate uridylyltransferase	8.014722	5.163009	2.51E-21	3.18E-19
CWC46_RS01805	*kdtX*	glycosyltransferase family 2 protein	8.063718	4.238021	1.26E-19	1.54E-17
CWC46_RS08340		glutamate-cysteine ligase	7.904938	3.456844	1.53E-19	1.80E-17
CWC46_RS13470	*srfB*	virulence protein SrfB	10.504050	3.094624	1.55E-19	1.80E-17
CWC46_RS13465	*srfC*	Virulence effector protein SrfC	10.153745	3.046067	2.49E-18	2.86E-16
CWC46_RS04070		class I adenylate cyclase	8.602088	4.326770	4.26E-18	4.81E-16

Locus tag	Gene	Product	logCPM	log2FC MOI1	PValue MOI1	padj MOI1
CWC46_RS13475		hypothetical protein	9.719238	2.833761	7.72E-16	8.46E-14
CWC46_RS07640	*apbE*	FAD:protein FMN transferase	8.512412	2.496250	1.49E-14	1.58E-12
CWC46_RS03605	*oxyR*	DNA-binding transcriptional regulator OxyR	7.956662	2.991932	1.13E-13	1.17E-11
CWC46_RS08390		3-deoxy-7-phosphoheptulonate synthase	6.133735	4.951394	1.03E-12	1.05E-10
CWC46_RS04470	*crp*	cAMP-activated global transcriptional regulator CRP	6.662197	5.779954	5.19E-12	5.16E-10
CWC46_RS00505	*rpoN*	RNA polymerase sigma-54 factor	6.590869	4.413928	6.57E-10	5.48E-08
CWC46_RS11420	*flgM*	anti-sigma-28 factor FlgM	5.269908	5.395807	1.26E-09	9.95E-08
CWC46_RS05635		glutathione synthase	6.270372	4.299086	2.06E-09	1.53E-07
CWC46_RS00950	*trkH*	Trk system potassium transporter TrkH	6.857487	4.159567	9.98E-09	6.25E-07
CWC46_RS12375	*sapF*	peptide ABC transporter ATP-binding protein SapF	4.719245	7.837456	1.71E-08	9.80E-07
CWC46_RS02180	*ntrB*	nitrogen regulation protein NR(II)	6.292934	3.229692	7.41E-08	3.06E-06
CWC46_RS02185	*ntrC*	sigma-54-dependent Fis family transcriptional regulator	6.628343	2.495368	9.29E-07	2.17E-05
CWC46_RS18950	*clpX*	ATP-dependent protease ATP-binding subunit ClpX	5.952751	3.824372	1.28E-06	2.73E-05
CWC46_RS03500		triose-phosphate isomerase	5.158205	5.249941	1.50E-06	3.05E-05
CWC46_RS07310	*cysH*	phosphoadenosine phosphosulfate reductase	5.399234	4.716191	2.25E-06	4.06E-05
CWC46_RS01830		glycosyltransferase family 1 protein	6.908378	2.013382	2.54E-06	4.42E-05
CWC46_RS05800	*thrB*	homoserine kinase	5.558213	3.422501	4.36E-06	6.82E-05
CWC46_RS07305	*cysI*	assimilatory sulfite reductase (NADPH) hemoprotein subunit	6.218618	3.448626	9.26E-06	1.24E-04
CWC46_RS07300	*cysJ*	NADPH-dependent assimilatory sulfite reductase flavoprotein subunit	6.063885	2.741004	1.03E-05	1.36E-04
CWC46_RS12395	*sapA*	peptide ABC transporter substrate-binding protein SapA	5.444007	4.291211	1.21E-05	1.53E-04
CWC46_RS13760	*rsxC*	electron transport complex subunit RsxC	5.597304	3.207741	1.34E-05	1.65E-04
CWC46_RS07330	*cysC*	adenylyl-sulfate kinase	5.158266	4.378136	7.92E-05	7.07E-04
CWC46_RS17860		NAD-dependent epimerase	6.747649	1.499182	1.44E-04	1.13E-03
CWC46_RS19675	*rppH*	RNA pyrophosphohydrolase	5.513617	3.025520	5.23E-04	3.24E-03

Locus tag	Gene	Product	logCPM	log2FC MOI1	PValue MOI1	padj MOI1
CWC46_RS18955	*clpP*	ATP-dependent Clp endopeptidase, proteolytic subunit ClpP	4.864441	3.154680	1.41E-03	7.34E-03
CWC46_RS18195		alpha-D-glucose phosphate-specific phosphoglucomutase	4.905083	2.537293	1.54E-03	7.81E-03
CWC46_RS03790	*gppA*	guanosine-5'-triphosphate,3'-diphosphate diphosphatase	6.390639	1.340632	1.71E-03	8.50E-03
CWC46_RS15835	*trxB*	thioredoxin-disulfide reductase	5.251284	2.741599	1.72E-03	8.56E-03
CWC46_RS05940	*apaH*	bis(5'-nucleosyl)-tetraphosphatase (symmetrical)	5.042499	3.286773	2.60E-03	1.17E-02
CWC46_RS21065	*rnfH*	RnfH family protein	4.431621	3.942610	3.10E-03	1.34E-02
CWC46_RS12380	*sapD*	peptide ABC transporter ATP-binding protein SapD	3.198529	7.762651	3.44E-03	1.46E-02
CWC46_RS00480	*kdsD*	KdsD/GutQ family sugar-phosphate isomerase	4.734949	3.491665	4.40E-03	1.76E-02
CWC46_RS07405	*gmhB*	D-glycero-beta-D-manno-heptose 1,7-bisphosphate 7-phosphatase	4.141783	3.522148	4.63E-03	1.83E-02
CWC46_RS05805		threonine synthase	5.170451	2.104981	5.66E-03	2.14E-02
CWC46_RS20990		efflux RND transporter periplasmic adaptor subunit	7.895340	0.989739	6.44E-03	2.36E-02
CWC46_RS13335		hypothetical protein	2.927120	7.487084	7.12E-03	2.55E-02
CWC46_RS11715	*lpxL*	lipid A biosynthesis lauroyl (or palmitoleoyl) acyltransferase	2.872916	7.485194	1.02E-02	3.38E-02
CWC46_RS01845	*rfaF*	ADP-heptose--LPS heptosyltransferase RfaF	3.827178	4.811780	1.14E-02	3.68E-02
CWC46_RS12385	*sapC*	peptide ABC transporter permease SapC	4.124912	3.513519	1.21E-02	3.86E-02
CWC46_RS13755	*rsxB*	electron transport complex subunit RsxB	2.787006	7.289306	1.28E-02	4.04E-02
CWC46_RS10480	*nuoI*	NADH-quinone oxidoreductase subunit NuoI	2.222977	6.742093	1.44E-02	4.38E-02
CWC46_RS18820	*apt*	adenine phosphoribosyltransferase	5.384006	1.985254	1.49E-02	4.51E-02
CWC46_RS22130	*rlmE*	23S rRNA (uridine(2552)-2'-O)-methyltransferase RlmE	2.859606	7.465785	1.51E-02	4.55E-02

Table S6. Serratia sp. *Serratia sp.* ATCC 39006 intergenic regions significantly enriched in transposon sequencing after jumbo phage PCH45 challenge.

Intergenic region	Start	End	Is downstream gene a hit?	Gene encoded downstream	Downstream gene locus tag	logCPM	log2FC MOI1	PValue MOI1	padj MOI1
Intergenic_nt2531759	2531759	2532048	yes	*fliC*, flagellin	CWC46_RS11570	10.545	5.258	3.43E-55	1.58E-52
Intergenic_nt2489250	2489250	2490575	yes	*flhD*, flagellar transcriptional regulator	CWC46_RS11345	15.242	4.620	3.99E-50	1.13E-47
Intergenic_nt2521995	2521995	2522143	yes	*fliL*, flagellar basal body-associated protein	CWC46_RS11530	8.564	5.341	4.31E-46	9.60E-44
Intergenic_nt2528669	2528669	2529008	yes	*fliF* and *fliE*	CWC46_RS11545 / CWC46_RS11550	10.291	4.714	1.36E-30	2.22E-28
Intergenic_nt2501551	2501551	2501780	yes	*flhB*, flagellar type III secretion system protein	CWC46_RS11400	8.707	3.847	4.37E-30	6.98E-28
Intergenic_nt2507020	2507020	2507178	yes	*flgA*, flagellar basal body P-ring formation protein	CWC46_RS11425	9.295	4.102	8.19E-29	1.25E-26
Intergenic_nt2514508	2514508	2514626	yes	*flgK*, flagellar hook-associated protein	CWC46_RS11475	9.103	3.933	9.59E-26	1.38E-23
Intergenic_nt2518829	2518829	2518876	yes	*fliQ*, flagellar biosynthetic protein	CWC46_RS11490	7.736	3.750	6.46E-24	9.13E-22
Intergenic_nt2510767	2510767	2510793	yes	*flgG*, flagellar basal-body rod protein	CWC46_RS11455	6.908	4.842	2.04E-22	2.78E-20
Intergenic_nt2516547	2516547	2516578	yes	*flgL*, flagellar hook-filament junction protein	CWC46_RS11480	6.417	6.993	5.90E-22	7.88E-20
Intergenic_nt2507593	2507593	2507622	yes	*flgB*, flagellar basal body rod protein	CWC46_RS11430	6.878	4.711	1.24E-21	1.62E-19
Intergenic_nt2508715	2508715	2508777	yes	*flgE*, flagellar hook protein	CWC46_RS11445	7.297	5.302	1.40E-21	1.80E-19
Intergenic_nt4705395	4705395	4706160	no	region between hypothetical protein and acetate-CoA ligase. US both	CWC46_RS21475 and CWC46_RS21480	10.043	3.120	2.47E-20	3.07E-18

Intergenic region	Start	End	Is downstream gene a hit?	Gene encoded downstream	Downstream gene locus tag	logCPM	log2FC MOI1	PValue MOI1	padj MOI1
Intergenic_nt2491511	2491511	2491636	yes	*motA*, flagellar motor stator protein MotA	CWC46_RS11355	8.592	3.245	1.49E-19	1.80E-17
Intergenic_nt2986379	2986379	2986615	yes	hypothetical protein (same operon as virulence factor *srfBC*)	CWC46_RS13475	7.531	2.883	5.86E-14	6.15E-12
Intergenic_nt129531	129531	129963	yes	*igaA*	CWC46_RS00620	7.850	2.507	5.58E-11	5.12E-09
Intergenic_nt2511577	2511577	2511705	yes	*flgH*, flagellar basal body L-ring protein	CWC46_RS11460	6.835	3.109	1.09E-09	8.83E-08
Intergenic_nt4754882	4754882	4755120	yes	*rfbB*, dTDP-glucose 4-6-dehydratase	CWC46_RS21685	8.460	2.096	6.54E-08	2.81E-06
Intergenic_nt2564684	2564684	2565571	yes	*mdoG*, glucan biosynthesis protein G	CWC46_RS11700	9.065	1.442	2.29E-06	4.11E-05
Intergenic_nt2548557	2548557	2548971	yes	*fliA*, RNA polymerase sigma factor	CWC46_RS11610	10.064	1.341	4.20E-06	6.63E-05
Intergenic_nt882303	882303	882634	yes	class I adenylate cyclase	CWC46_RS04070	5.711	3.069	7.90E-06	1.10E-04
Intergenic_nt3107086	3107086	3107157	yes	*rssB*, two-component system response regulator	CWC46_RS13965	5.296	3.096	9.28E-05	8.00E-04
Intergenic_nt2509987	2509987	2510007	yes	*flgF*, flagellar basal body rod protein	CWC46_RS11450	3.979	8.589	3.63E-04	2.40E-03
Intergenic_nt2508028	2508028	2508039	yes	*flgD*, flagellar hook assembly protein	CWC46_RS11440	5.515	3.397	6.56E-04	3.88E-03
Intergenic_nt969004	969004	969324	yes	*crp*, cAMP-activated global transcriptional regulator CRP	CWC46_RS04470	6.604	1.700	8.75E-04	4.89E-03
Intergenic_nt2490927	2490927	2490931	yes	*flhC*, flagellar transcriptional regulator	CWC46_RS11350	3.685	4.783	2.17E-03	1.01E-02
Intergenic_nt2506215	2506215	2506353	yes	*flgM*, anti-sigma-28 factor FlgM	CWC46_RS11420	4.352	4.005	3.29E-03	1.41E-02

Intergenic region	Start	End	Is downstream gene a hit?	Gene encoded downstream	Downstream gene locus tag	logCPM	log2FC MOI1	PValue MOI1	padj MOI1
Intergenic_nt4882882	4882882	4882987	no	upstream glutathione S-transferase family protein	CWC46_RS22330	2.941	5.144	7.33E-03	2.61E-02
Intergenic_nt3823173	3823173	3823239	no	upstream RND efflux system, outer membrane lipoprotein, NodT family CDS	CWC46_RS17460	2.473	7.027	8.73E-03	2.99E-02
Intergenic_nt3853349	3853349	3853492	no	upstream DeoR/GlpR transcriptional regulator	CWC46_RS17575	2.808	5.758	9.86E-03	3.29E-02
Intergenic_nt1602300	1602300	1602324	yes	*cysC*, adenylyl-sulfate kinase	CWC46_RS07330	2.827	7.416	1.14E-02	3.68E-02
Intergenic_nt3292089	3292089	3292129	no	region between sensor domain-containing diguanylate cyclase and phosphoprotein phosphatase, ds both genes	CWC46_RS14985 and CWC46_RS14990	2.402	6.953	1.16E-02	3.74E-02
Intergenic_nt2521504	2521504	2521508	yes	*fliM*, flagellar motor switch protein	CWC46_RS11510	2.692	7.233	1.30E-02	4.07E-02
Intergenic_nt1552554	1552554	1552738	no	inorganic diphosphatase	CWC46_RS07135	2.401	6.953	1.32E-02	4.12E-02

Table S7. Genomic context, localisation of mutant loci identified in Tn-seq screen.

Locus tag	Gene	Distribution	Biological function			Location
CWC46_RS11475	*flgK*	operon_9	flagella assembly / cell motility			extracellular
CWC46_RS11480	*flgL*	operon_9	flagella assembly / cell motility			extracellular
CWC46_RS11550	*fliE*	sag_8	flagella assembly / cell motility			outer membrane
CWC46_RS11485	*fliR*	operon_10	flagella assembly / cell motility			inner membrane
CWC46_RS11490	*fliQ*	operon_10	flagella assembly / cell motility			inner membrane
CWC46_RS11350	*flhC*	operon_5	flagella assembly / cell motility	regulation of transcription, DNA-templated		cytosol
CWC46_RS11345	*flhD*	operon_5	flagella assembly / cell motility	regulation of transcription, DNA-templated		cytosol
CWC46_RS11495	*fliP*	operon_10	flagella assembly / cell motility			inner membrane
CWC46_RS11575		operon_12	flagella assembly / cell motility			unknown
CWC46_RS11530	*fliI*	operon_11	flagella assembly / cell motility			cytosol
CWC46_RS11440	*flgD*	operon_9	flagella assembly / cell motility			
CWC46_RS11400	*flhB*	operon_7	flagella assembly / cell motility			inner membrane
CWC46_RS11445	*flgE*	operon_9	flagella assembly / cell motility			extracellular
CWC46_RS11610	*fliA*	oeo_14	flagella assembly / cell motility	regulation of transcription, DNA-templated		cytosol
CWC46_RS11425	*flgA*	sag_7	flagella assembly / cell motility			periplasm
CWC46_RS21680	*rfbA*	operon_18	lipopolysaccharide biosynthesis			cytosol
CWC46_RS11510	*fliM*	operon_10	flagella assembly / cell motility			inner membrane

Locus tag	Gene	Distribution	Biological function			Location
CWC46_RS11540	*fliG*	operon_11	flagella assembly / cell motility			inner membrane
CWC46_RS11570	*fliC*	operon_12	flagella assembly / cell motility			extracellular
CWC46_RS11525	*fliJ*	operon_11	flagella assembly / cell motility			inner membrane
CWC46_RS11545	*fliF*	operon_11	flagella assembly / cell motility			inner membrane
CWC46_RS11565	*fliD*	oeo_13	flagella assembly / cell motility			extracellular
CWC46_RS11405	*flhA*	operon_7	flagella assembly / cell motility			inner membrane
CWC46_RS21670	*rfbC*	operon_18	lipopolysaccharide biosynthesis			cytosol
CWC46_RS11460	*flgH*	operon_9	flagella assembly / cell motility			outer membrane
CWC46_RS11360	*motB*	operon_6	flagella assembly / cell motility			inner membrane
CWC46_RS21675	*rfbD*	operon_18	lipopolysaccharide biosynthesis			cytosol
CWC46_RS11435	*flgC*	operon_9	flagella assembly / cell motility			periplasm
CWC46_RS11415	*flgN*	operon_8	flagella assembly / cell motility			cytosol
CWC46_RS11505	*fliN*	operon_10	flagella assembly / cell motility			inner membrane
CWC46_RS11465	*flgI*	operon_9	flagella assembly / cell motility			periplasm
CWC46_RS11355	*motA*	operon_6	flagella assembly / cell motility			inner membrane
CWC46_RS21685	*rfbB*	operon_18	lipopolysaccharide biosynthesis			cytosol
CWC46_RS11520	*fliK*	operon_11	flagella assembly / cell motility			inner membrane
CWC46_RS11535	*fliH*	operon_11	flagella assembly / cell motility			cytosol

Locus tag	Gene	Distribution	Biological function			Location
CWC46_RS11705	*mdoH*	operon_13	Glucans biosynthesis			inner membrane
CWC46_RS13965	*rssB*	oeo_16	phosphorelay signal transduction system	regulation of transcription	signal transduction	cytosol
CWC46_RS11500	*fliO*	operon_10	flagella assembly / cell motility			inner membrane
CWC46_RS11515	*fliL*	operon_10	flagella assembly / cell motility			inner membrane
CWC46_RS11700	*mdoG*	operon_13	Glucans biosynthesis			periplasm
CWC46_RS11455	*flgG*	operon_9	flagella assembly / cell motility			outer membrane
CWC46_RS11450	*flgF*	operon_9	flagella assembly / cell motility			outer membrane
CWC46_RS00620	*igaA*	oeo_3	signal transduction			inner membrane
CWC46_RS11470	*flgJ*	operon_9	flagella assembly / cell motility			periplasm
CWC46_RS11430	*flgB*	operon_9	flagella assembly / cell motility			periplasm
CWC46_RS13960	*galU*	sag_10	lipopolysaccharide biosynthesis			cytosol
CWC46_RS01805	*kdtX*	oeo_5	lipopolysaccharide biosynthesis			unknown
CWC46_RS08340		sag_6	glutathione biosynthetic process			cytosol
CWC46_RS13470	*srfB*	operon_15	virulence factor			unknown
CWC46_RS13465	*srfC*	operon_15	virulence factor			unknown
CWC46_RS04070		sag_2	signal transduction			cytosol
CWC46_RS13475		operon_15	virulence factor			unknown
CWC46_RS07640	*apbE*	sag_5	Other			inner membrane
CWC46_RS03605	*oxyR*	sag_1	regulation of transcription, DNA-templated			cytosol
CWC46_RS08390		oeo_11	amino acid biosynthetic process			cytosol

Locus tag	Gene	Distribution	Biological function			Location
CWC46_RS04470	*crp*	sag_3	regulation of transcription, DNA-templated	signal transduction		cytosol
CWC46_RS00505	*rpoN*	oeo_2	regulation of transcription, DNA-templated			cytosol
CWC46_RS11420	*flgM*	operon_8	flagella assembly / cell motility	regulation of transcription, DNA-templated		cytosol
CWC46_RS05635		oeo_8	glutathione biosynthetic process			cytosol
CWC46_RS00950	*trkH*	oeo_4	transport			inner membrane
CWC46_RS12375	*sapF*	operon_14	transport			inner membrane
CWC46_RS02180	*ntrB*	operon_2	phosphorelay sensor kinase activity	regulation of transcription, DNA-templated	signal transduction	unknown
CWC46_RS02185	*ntrC*	operon_2	phosphorelay signal transduction system		signal transduction	cytosol
CWC46_RS18950	*clpX*	operon_17	proteolysis			cytosol
CWC46_RS03500		oeo_6	Other			cytosol
CWC46_RS07310	*cysH*	operon_4	amino acid biosynthetic process			cytosol
CWC46_RS01830		operon_1	lipopolysaccharide biosynthesis			unknown
CWC46_RS05800	*thrB*	operon_3	amino acid biosynthetic process			cytosol
CWC46_RS07305	*cysI*	operon_4	amino acid biosynthetic process	Energy production and conversion		cytosol
CWC46_RS07300	*cysJ*	operon_4	amino acid biosynthetic process			cytosol
CWC46_RS12395	*sapA*	operon_14	transport			periplasm
CWC46_RS13760	*rsxC*	operon_16	Energy production and conversion			inner membrane
CWC46_RS07330	*cysC*	oeo_10	amino acid biosynthetic process			cytosol
CWC46_RS17860		sag_12	Other			unknown
CWC46_RS19675	*rppH*	oeo_19	Translation, ribosomal structure and biogenesis			cytosol

Locus tag	Gene	Distribution	Biological function			Location
CWC46_RS18955	*clpP*	operon_17	proteolysis			cytosol
CWC46_RS18195		oeo_17	Other			unknown
CWC46_RS03790	*gppA*	oeo_7	signal transduction			unknown
CWC46_RS15835	*trxB*	sag_11	Other			cytosol
CWC46_RS05940	*apaH*	oeo_9	Other			unknown
CWC46_RS21065	*rnfH*	oeo_21	defence			unknown
CWC46_RS12380	*sapD*	operon_14	transport			inner membrane
CWC46_RS00480	*kdsD*	oeo_1	lipopolysaccharide biosynthesis			unknown
CWC46_RS07405	*gmhB*	sag_4	lipopolysaccharide biosynthesis			cytosol
CWC46_RS05805		operon_3	amino acid biosynthetic process			unknown
CWC46_RS20990		oeo_20	transport			inner membrane
CWC46_RS13335		oeo_15	Other			unknown
CWC46_RS11715	*lpxL*	sag_9	lipopolysaccharide biosynthesis			inner membrane
CWC46_RS01845	*rfaF*	operon_1	lipopolysaccharide biosynthesis			cytosol
CWC46_RS12385	*sapC*	operon_14	transport			inner membrane
CWC46_RS13755	*rsxB*	operon_16	Energy production and conversion			inner membrane
CWC46_RS10480	*nuoI*	oeo_12	Energy production and conversion			inner membrane
CWC46_RS18820	*apt*	oeo_18	Other			cytosol
CWC46_RS22130	*rlmE*	oeo_22	Translation, ribosomal structure and biogenesis			cytosol

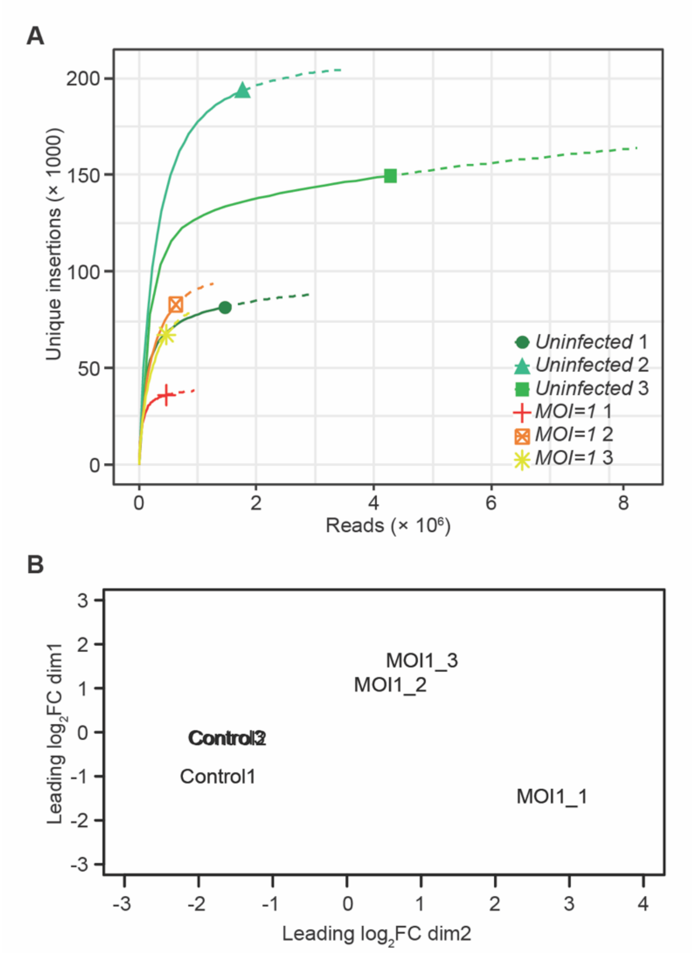

Figure S1. A. Rarefaction curves used to assess sequencing depth during Tn-seq. Rarefaction curves were generated in R using the iNEXT package. Solid lines show interpolated data, while dashed lines show extrapolated (predicted) data, to simulate additional sequencing depth for uninfected and infected libraries. **B.** PCA plot between sample replicates based on the top 1000 hits ($log_2(FC)>0.5$ and $P_{adj}<0.05$).

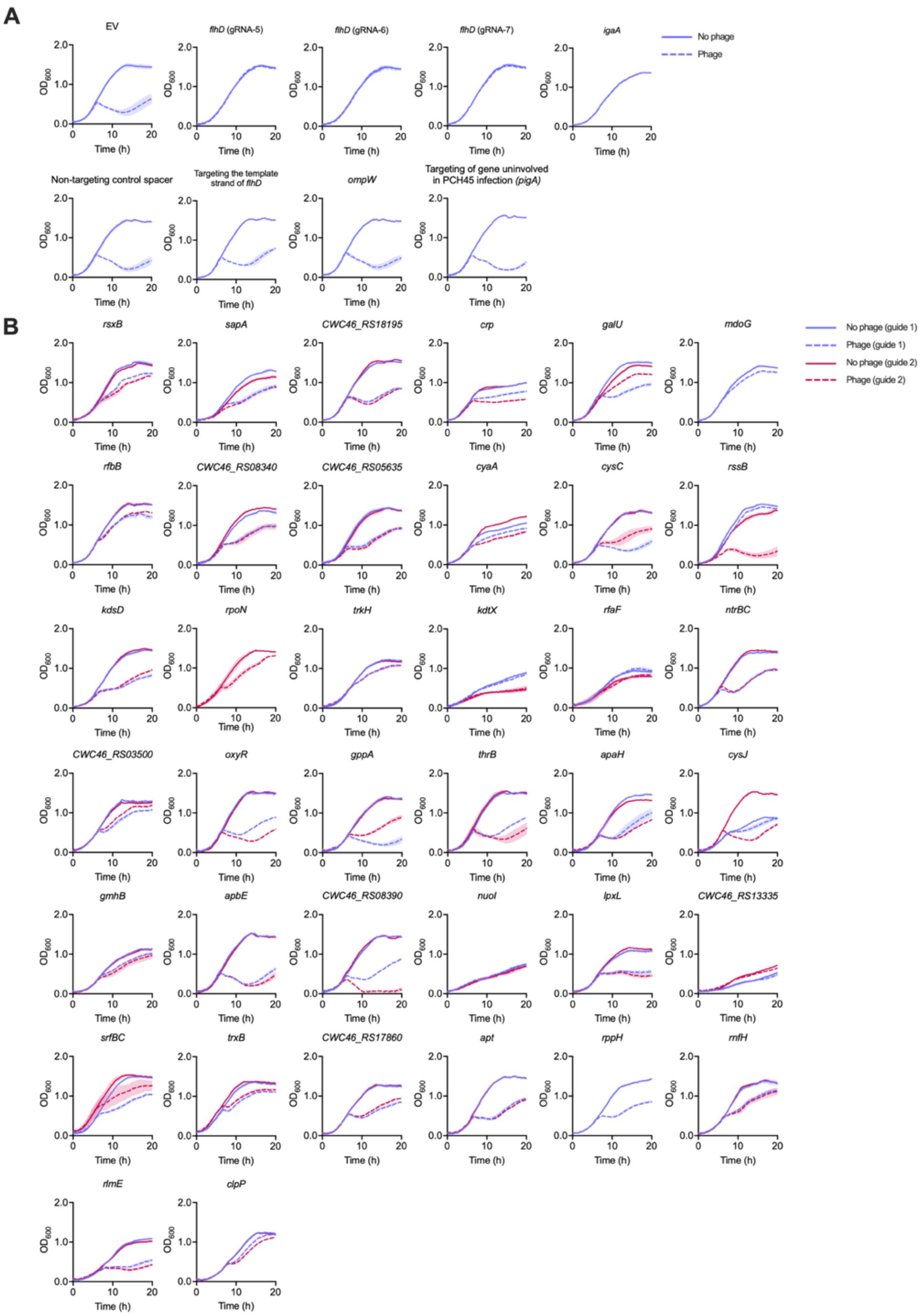

Figure S2. Liquid growth curves of all CRISPRi knockdown strains tested. A. Growth curve results for CRISPRi control strains and **B.** CRISPRi silenced host genes identified to be involved in jumbo phage infection by Tn-seq analysis**.** *Serratia* growth (OD_{600}) was monitored with (dashed lined) and without (solid line) PCH45 phage challenge at an moi=0. 01. Both single-guide RNAs (sgRNAs) designed for each genes are shown (purple = first sgRNA design, red = second sgRNA design); some genes may only have 1 guide, or more than 2 guides (i.e. *flhD* has 4 sgRNA designs, as shown in panel A). Data shown is the mean (lines) of biological triplicates +/- SD (shading) (EV = 21 biological replicates, 3 per plate). See Table S2 and S3 for CRISPRi sgRNA construct design.

A

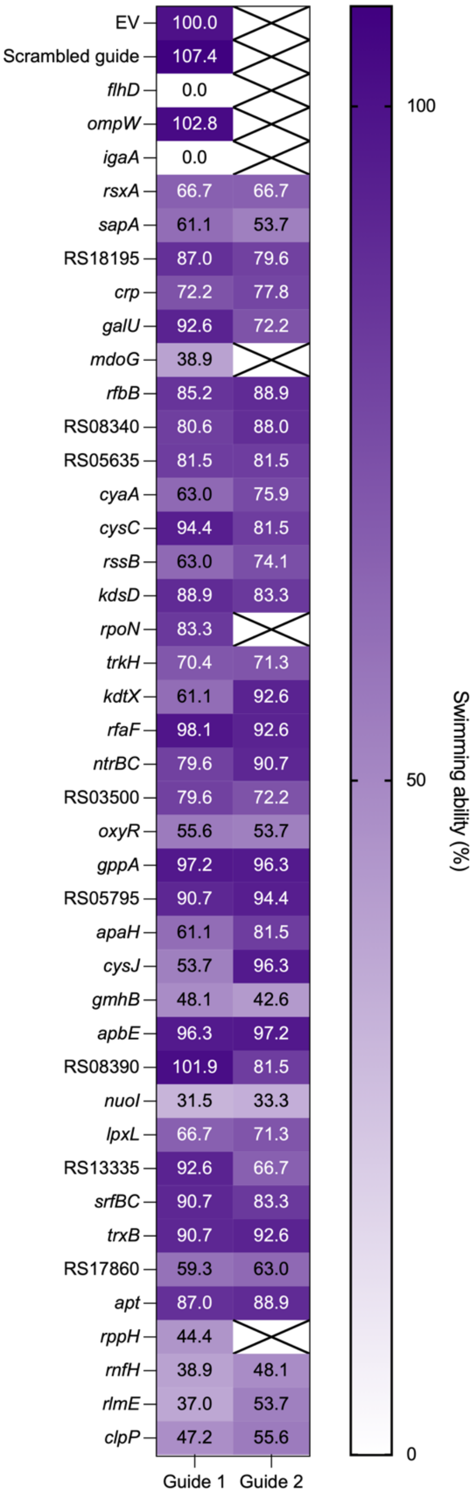

Figure S3. Swimming assay results for all CRISPRi knockdown strains tested. A. Both single-guide RNAs (sgRNAs) designed for each gene are shown (some genes may only have 1 guide, X= no second guide). Values in percentage (%) are based on the average of three biological triplicates. The empty vector (EV) strain has nine biological replicates as 3 TSA plates were used to assess all sgRNA constructs. See Table S2 and S3 for CRISPRi sgRNA construct design.

References

1. Thoma S, Schobert M. An improved *Escherichia coli* donor strain for diparental mating. *FEMS Microbiol Lett.* 2009;294(2):127-32.

2. Thomson NR, Crow MA, McGowan SJ, Cox A, Salmond GP. Biosynthesis of carbapenem antibiotic and prodigiosin pigment in *Serratia* is under quorum sensing control. *Mol Microbiol.* 2000;36(3):539-56.

3. Malone LM, Warring SL, Jackson SA, Warnecke C, Gardner PP, Gumy LF, et al. A jumbo phage that forms a nucleus-like structure evades CRISPR-Cas DNA targeting but is vulnerable to type III RNA-based immunity. *Nat Microbiol*. 2020;5(1):48-55.

4. Mahler M, Malone LM, van den Berg DF, Smith LM, Brouns SJJ, Fineran PC. An OmpW-dependent T4-like phage infects *Serratia* sp. ATCC 39006. *Microb Genom*. 2023;9(3).

5. Evans TJ, Crow MA, Williamson NR, Orme W, Thomson NR, Komitopoulou E, et al. Characterization of a broad-host-range flagellum-dependent phage that mediates high-efficiency generalized transduction in, and between, *Serratia* and *Pantoea*. *Microbiology* (Reading). 2010;156(Pt 1):240-7.

6. Malone LM, Hampton HG, Morgan XC, Fineran PC. Type I CRISPR-Cas provides robust immunity but incomplete attenuation of phage-induced cellular stress. *Nucleic Acids Res*. 2022;50(1):160-74.

7. Patterson, A. G., Jackson, S. A., Taylor, C., Evans, G. B., Salmond, G. P. C., Przybilski, R., Staals, R. H. J., & Fineran, P. C. (2016). Quorum Sensing Controls Adaptive Immunity through the Regulation of Multiple CRISPR-Cas Systems. *Molecular cell*, *64*(6), 1102–1108.
